# Identification and characterization of the *Remorin* gene family in *Saccharum* and the involvement of ScREM1.5e-1/-2 in SCMV infection on sugarcane

**DOI:** 10.3389/fpls.2024.1365995

**Published:** 2024-02-23

**Authors:** Zongtao Yang, Guangyuan Cheng, Quanxin Yu, Wendi Jiao, Kang Zeng, Tingxu Luo, Hai Zhang, Heyang Shang, Guoqiang Huang, Fengji Wang, Ying Guo, Jingsheng Xu

**Affiliations:** ^1^ Key Laboratory of Sugarcane Biology and Genetic Breeding, Ministry of Agriculture and Rural Affairs, National Engineering Research Center for Sugarcane, College of Agriculture, Fujian Agriculture and Forestry University, Fuzhou, Fujian, China; ^2^ Key Laboratory of Ministry of Education for Genetics, Breeding and Multiple Utilization of Crops, College of Crop Science, Fujian Agriculture and Forestry University, Fuzhou, China; ^3^ Fujian Key Laboratory of Subtropical Plant Physiology and Biochemistry, Fujian Institute of Subtropical Botany, Xiamen, Fujian, China

**Keywords:** sugarcane, Remorin, gene family, virus, VPg

## Abstract

**Introduction:**

Remorins (REMs) are plant-specific membrane-associated proteins that play important roles in plant–pathogen interactions and environmental adaptations. Group I REMs are extensively involved in virus infection. However, little is known about the *REM* gene family in sugarcane (*Saccharum* spp. hyrid), the most important sugar and energy crop around world.

**Methods:**

Comparative genomics were employed to analyze the *REM* gene family in *Saccharum spontaneum*. Transcriptomics or RT-qPCR were used to analyze their expression files in different development stages or tissues under different treatments. Yeast two hybrid, bimolecular fluorescence complementation and co-immunoprecipitation assays were applied to investigate the protein interaction.

**Results:**

In this study, 65 *REMs* were identified from *Saccharum spontaneum* genome and classified into six groups based on phylogenetic tree analysis. These REMs contain multiple *cis*-elements associated with growth, development, hormone and stress response. Expression profiling revealed that among different *SsREMs* with variable expression levels in different developmental stages or different tissues. A pair of alleles, *ScREM1.5e-1/-2*, were isolated from the sugarcane cultivar ROC22. *ScREM1.5e-1/-2* were highly expressed in leaves, with the former expressed at significantly higher levels than the latter. Their expression was induced by treatment with H_2_O_2_, ABA, ethylene, brassinosteroid, SA or MeJA, and varied upon *Sugarcane mosaic virus* (SCMV) infection. ScREM1.5e-1 was localized to the plasma membrane (PM), while ScREM1.5e-2 was localized to the cytoplasm or nucleus. ScREM1.5e-1/-2 can self-interact and interact with each other, and interact with VPgs from SCMV, *Sorghum mosaic virus*, or *Sugarcane streak mosaic virus*. The interactions with VPgs relocated ScREM1.5e-1 from the PM to the cytoplasm.

**Discussion:**

These results reveal the origin, distribution and evolution of the *REM* gene family in sugarcane and may shed light on engineering sugarcane resistance against sugarcane mosaic pathogens.

## 1 Introduction

Membrane rafts are ordered liquid nanodomains formed by lateral aggregation of sterols, sphingolipids and specific proteins on the cell plasma membrane (PM) and are important platforms for signal transduction (Mongrand et al., 2010; Otero et al., 2016; Perraki et al., 2018). Remorins (REMs), first discovered in tomato and potato, are a class of membrane raft-associated proteins unique to land plants (Farmer et al., 1989; Jacinto et al., 1993; Reymond et al., 1996). The REM protein consists of a highly variable N-terminal domain (PF03766) and a conserved C-terminal domain (PF03763) containing a coiled-coil motif and an anchor (RemCA) (Raffaele et al., 2007; Perraki et al., 2012; Yue et al., 2014; Badawi et al., 2019; Wang et al., 2022; Li et al., 2023). Although REM contains no transmembrane domain, the C-terminal coiled-coil motif and RemCA can distribute REM to the PM (Perraki et al., 2014). In addition, the palmitoylation of cysteine residues in the C-terminal domain and the phosphorylation of serine residues in the N-terminal domain contribute to the PM localization of REM proteins (Marín and Ott, 2012; Marín et al., 2012; Konrad et al., 2014; Fu et al., 2018; Gouguet et al., 2021; Legrand et al., 2023). Based on their structural characteristics, REM proteins are divided into 6 main groups (Raffaele et al., 2007). *REM* gene families have been found in many plant species, including poplar (*Populus trichocarpa*), Arabidopsis (*Arabidopsis thaliana*), tomato (*Solanum lycopersicum*), rice (*Oryza sativa*), wheat (*Triticum aestivum*), maize (*Zea mays*) and foxtail millet (*Setaria italica*) (Raffaele et al., 2007; Badawi et al., 2019; Wang et al., 2022; Li et al., 2023). Sugarcane (*Saccharum* spp. hybrid) is the most important sugar and energy crop in China and worldwide, but the *REM* gene family has not been reported for this plant.

*REMs* are involved in plant growth and development and in responding to biotic or abiotic stresses (Bray, 2002; Reddy et al., 2002; Nohzadeh Malakshah et al., 2007; Checker and Khurana, 2013; Li et al., 2014; Cai et al., 2018; Fu et al., 2018; Cheng et al., 2020). Overexpression of remorin *GSD1* (from Group 6) in rice increased callose accumulation in the plasmodesmata and inhibited the transport of assimilates to grains (Gui et al., 2014). Overexpression of *SlREM1* in tomato stimulated fruit ripening and promoted ethylene production and lycopene accumulation (Cai et al., 2018). The literature has shown that *REMs* respond to biological stresses such as infection by viruses (Raffaele et al., 2009; Jarsch and Ott, 2011; Perraki et al., 2012; Wu et al., 2013; Perraki et al., 2014; Fu et al., 2018; Perraki et al., 2018; Sasaki et al., 2018), bacteria (Lefebvre et al., 2010; Tóth et al., 2012; Bozkurt et al., 2014; Liang et al., 2018) and fungi (Son et al., 2015; Jamann et al., 2016; Vilakazi et al., 2017); abiotic stresses such as drought, salt and low temperature (Nohzadeh Malakshah et al., 2007; Li et al., 2012; Checker and Khurana, 2013; Yue et al., 2014; Badawi et al., 2019; Zhang et al., 2020; Gouguet et al., 2021; Wang et al., 2022); and hormones (Li et al., 2014; Gui et al., 2016; Badawi et al., 2019; Huang et al., 2019; Gouguet et al., 2021; Wang et al., 2022). However, Group 1 *REMs* are involved mainly in the response to virus infection. For example, OsREM1.4 of rice or NbREM1 of *Nicotiana benthamiana* interact with the movement protein NSvc4 of *Rice stripe virus* (RSV), and overexpression of *OsREM1.4* or *NbREM1* inhibited RSV infection (Fu et al., 2018). Overexpression of tomato *StREM1.3* inhibited the 30 kD movement protein of *Tobacco mosaic virus* (TMV) and the intercellular movement of *Potato virus Y* (PVY) HC-Pro (Perraki et al., 2014). In tomato, StREM1.3 interacts with TGBp1, a movement protein of *Potato virus X* (PVX), and overexpression of *StREM1.3* impairs PVX infection by gating the plasmodesmata (Rajamäki and Valkonen, 2009; Perraki et al., 2012, 2014). REMs can oligomerize into a scaffolding structure and cooperate with flotillins to stabilize large-scale membrane conformations and the actin cytoskeleton (Liang et al., 2018; Su et al., 2023). The oligomerization of REMs also reduced plasma membrane fluidity and plasmodesmata permeability to impair CMV infection (Huang et al., 2019). In Arabidopsis, overexpression of *REM1.2* or *REM1.3* inhibited TuMV infection (Cheng et al., 2020).

Sugarcane mosaic disease seriously threatens sugarcane production. The main causative agents are *Sugarcane mosaic virus* (SCMV; *Potyvirus*), *Sorghum mosaic virus* (SrMV, *Potyvirus*) and *Sugarcane streak mosaic virus* (SCSMV; *Poacevirus*), all belonging to the *Potyviridae* family (Xu et al., 2008; Dong et al., 2017; Yao et al., 2017; Akbar et al., 2020, 2021; Hincapie et al., 2021). These 3 pathogens are single-stranded positive RNA viruses with a genome length of approximately 10 kb that encode 2 polyproteins, which are eventually hydrolyzed into 11 mature proteins: P1, HC-Pro, P3, P3N-PIPO, 6K1, CI, 6K2, NIa-Pro, VPg, NIb and CP (Ward and Shukla, 1991; Riechmann et al., 1992; Hall et al., 1998; Urcuqui-Inchima et al., 2001; Xu et al., 2010; Li et al., 2011; Olspert et al., 2015; Wang, 2015; Olspert et al., 2016; Cheng et al., 2017; Cui et al., 2017). Among these viral proteins, VPg is involved in viral replication, translation, movement and counteraction against host RNAi through interactions with other viral proteins and host proteins (Cotton et al., 2009; Huang et al., 2010; Cheng and Wang, 2017; Li and Wang, 2018; Cheng et al., 2020; Zhai et al., 2021). For example, TuMV utilizes VPg to interact with SGS3, REM1.2 or NbNdhM to mediate the degradation of SGS3 and REM1.2 via autophagy, the 26S ubiquitin proteasome system, or the perinuclear chloroplast aggregation of NbNdhM, respectively, to promote infection (Cheng and Wang, 2017; Cheng et al., 2020; Zhai et al., 2021). However, whether sugarcane Group 1 REMs interact with the VPgs of these three viruses (SCMV, SrMV and SCSMV) has not been determined.

In this study, members of the REM family were identified in sugarcane for the first time, and their phylogeny, chromosomal localization, physical and chemical properties, gene structure, conserved mods, *cis*-acting elements of promoters, and collinearity within and between species were analyzed. The expression of *REM* gene family members in different tissues and across leaf gradients was subsequently analyzed. Because Group 1 *REMs* mainly respond to viral infection, we cloned a pair of *REM1* alleles from the sugarcane cultivar ROC22 and designated them *ScREM1.5e-1/-2* based on phylogenetic tree analysis. The expression levels and subcellular localization of *REM1* alleles in different sugarcane tissues were analyzed, and the expression patterns of *REM1* alleles in response to SCMV infection were analyzed. The interactions between ScREM1.5e-1/-2 and the VPg proteins of SCMV, SCSMV, and SrMV were investigated, and whether ScREM1.5e-1 or ScREM1.5e-2 self-interact or interact with each other was evaluated. Finally, the expression levels of *ScREM1.5e-1/-2* under NaCl, PEG, H_2_O_2_, ABA, ETH, BR, SA and MeJA stress were analyzed. The aim of this study was to lay a foundation for the functional study of the *REM* genes in sugarcane and to provide potential molecular targets for engineering sugarcane resistance against sugarcane mosaic pathogens.

## 2 Materials and methods

### 2.1 Plant materials and methods

Tissue-cultured sugarcane cultivar ROC22 plantlets were cultured in a greenhouse with a 14/10-h light/dark cycle at 28°C and were individually inoculated with SCMV at the 5–6 leaf stage, as previously described (Zhang et al., 2019; Yang et al., 2021). The mock-inoculated ROC22 plantlets with 0.01 M phosphate buffer (pH 7.0) were used as the negative control. The inoculated or mock-inoculated leaves were sampled at 0 h, 6 h, 12 h, 24 h, 48 h, 5 d, 8 d and 14 d post-inoculation. For abiotic treatments, ROC22 plantlets of were grown in water for one week and then transferred to conical tubes for the following eight different treatments with a 16 h light/8 h dark cycle at 28°C. The plantlets were treated with 250 mM NaCl, 25% PEG (polyethylene glycol), 10 mM H_2_O_2_ (Su et al., 2014), 100 µM ABA, 400 mg/L ethylene solution (Chen et al., 2019) (Coolaber, Beijing, China), 25 mg/L BR (Wang et al., 2020), 5 mM SA, or 25 µM MeJA (methyl jasmonate) in 0.1% (v/v) ethanol, 0.05% (v/v) Tween-20, and were incubated for 0 h, 6 h, 12 h, 24 h and 48 h. After the treatments, three sugarcane plantlets per time point were sampled, frozen in liquid nitrogen immediately and kept at −80°C before use. Tissue samples from the leaves, roots and stem piths of 10-month-old healthy plants of ROC22 were also collected.

### 2.2 Identification, chromosomal localization and duplication events of the *REM* gene family

The genome sequence of *Saccharum spontaneum* was obtained from published genomic information (http://www.life.illinois.edu/ming/downloads/Spontaneum_genome) (Zhang et al., 2018). A Hidden Markov Model (HMM) of REM (Remorin_N: PF03766; Remorin_C: PF03763) was obtained from the Pfam database (http://pfam.xfam.org/) (Mistry et al., 2021) and was used to identify the *REM* genes in *S. spontaneum*. The gff3 files for *S. spontaneum* were downloaded (Zhang et al., 2018) to acquire the length and location information for the *REM* genes, then TBtools software was used to visualize the chromosomal locations of the *REM* genes. The chromosome color was set to black with the remaining parameters set to the default values. MCScanX software was used to analyze the replication pattern of the *REM* gene family with the parameters set to the default values (Wang et al., 2012).

### 2.3 Phylogenetic and physicochemical properties and subcellular localization

The phylogenetic tree of REM was constructed by using MEGA6 version with the neighbor−joining method and 1000 bootstrap replicates. The physicochemical properties of the REM protein, including the number of encoded amino acids, molecular weight, isoelectric point, instability index, aliphatic index and grand average of hydropathicity, were predicted by the ProtParam tool of ExPASy (https://web.expasy.org/protparam/) (Gasteiger et al., 2003). The Cell-PLoc 2.0 (http://www.csbio.sjtu.edu.cn/bioinf/Cell-PLoc-2/) website was used to predict the subcellular localization of REM proteins (Chou and Shen, 2008).

### 2.4 Motif and gene structure analysis

The *S. spontaneum REM* gene family motif information was acquired from the MEME website (https://meme-suite.org/meme/doc/meme.html) with the parameter was set to show 17 conserved motifs and the rest set as the default values (Bailey et al., 2009). The exon−intron structure information of the *REM* gene family was acquired from the gff3 files of *S. spontaneum*. The visualization of conserved motifs or gene structure of the *REM* gene family was conducted using TBtools software (Chen et al., 2020).

### 2.5 Synteny and homologous gene pairs

MCScanX software was used to analyze the synteny of the *REM* gene family with the parameters set to the default values (Wang et al., 2012). TBtools software was used to generate a synteny map within the genomes of *S. spontaneum*, sorghum (*Sorghum bicolor*), maize (*Zea mays*), wheat (*Triticum aestivum*), barley (*Hordeum vulgare*), and rice (*Oryza sativa*). The genome information of sorghum, maize, wheat, barley, and rice was acquired from Ensembl Plants (https://plants.ensembl.org/index.html). TBtools software was used to calculate the values of nonsynonymous (Ka)/synonymous (Ks) between homologous gene pairs based on the synteny relationship (Chen et al., 2020).

### 2.6 *Cis*-element analysis of the *REM* genes

The upstream 2,000 bp promoter sequences of the *REM* gene family members were acquired from the databases of *S. spontaneum* genome. The prediction of the *cis*-elements in the *REM* gene family was conducted through the PlantCARE online website (http://bioinformatics.psb.ugent.be/webtools/plantcare/html/) (Lescot et al., 2002), while the visualization of the *cis*-elements was conducted with TBtools software (Chen et al., 2020).

### 2.7 Expression profiles of *REM* gene families in various tissues

Sugarcane tissue-specific expression data included leaf and stem data at the seedling stage (35 d), early maturity stage (9 months) and maturity stage (12 months). Materials were taken from SES2086 sugarcane plants for 11 d. Leaves were divided into 15 segments from the base to the tip (a total of 15 cm); each segment was 1 cm long, and RNA was extracted from each leaf segment. For the leaf segment model, three representative regions of each material were taken. All *SsREMs* FPKMs (fragments per kilobase of transcript per million segments mapped) were used to construct heatmaps and perform cluster analysis through TBtools.

### 2.8 Total RNA extraction, cDNA first-strand synthesis and real-time quantitative PCR

TRIzol was used to isolate total RNA (Connolly et al., 2006). The Primer-Script^®^ RT−PCR Kit (TaKaRa Biotechnology, Dalian, China) was used to synthesize first-strand cDNA. For the real-time quantitative PCR (RT-qPCR) analysis, three technical replicates were completed for each sample with *25S rRNA* and *eEF-1a* were used as internal references (Iskandar et al., 2004; Guo et al., 2014; Ling et al., 2014). The specificity of each primer pair were confirmed by the melting curve analysis. The relative gene expression was calculated by using the 2^−ΔΔCt^ method in triplicate and presented as the means ± standard error.

### 2.9 Expression pattern of *ScREM1.5e-1/-2* in sugarcane

The ChamQ Universal SYBR qPCR Master Mix (Vazyme, Shanghai, China) was used to determine the expression patterns of *ScREM1.5e-1/-2* by RT-qPCR with the sugarcane cDNA using as the templates, and *25S rRNA* and *eEF-1a* were used as internal references (Iskandar et al., 2004; Guo et al., 2014; Ling et al., 2014) (**Supplementary Table 1**). The primers ScREM1.5e-1/-2-qPCR (**Supplementary Table 1**) were designed by the Primer Premier 5.0 software. RT−qPCR assays were conducted on the An ABI 7500 Real-time PCR System (Applied Biosystems, Foster City, CA, USA). The relative gene expression was calculated by using the 2^−ΔΔCt^ method in triplicate and presented as the means ± standard error.

### 2.10 Plasmid construction

The primers used for plasmid generation are listed in **Supplementary Table 1**. For the Yeast two hybrid (Y2H) assays, the Y2H vectors and all DNA fragments were individually ligated via *Sfi* I sites. SCMV-/SCSMV-/SrMV-VPg or ScREM1.5e-1/-2 were individually cloned into the bait vector pGBKT7, and ScREM1.5e-1/-2 were individually cloned into the prey vector pGADT7. For the transient protein expression and bimolecular fluorescence complementation (BiFC) assays, all plasmids were constructed using Gateway technology as previously described (Cheng et al., 2020). All the plasmids generated in the present study were verified by sequencing.

### 2.11 Protein interactions as determined by Y2H, BiFC and Co-immunoprecipitation assays

For the Y2H assay, the Matchmaker Gold Yeast Two-Hybrid System (Clontech, Mountain View, CA, USA) was used according to the manufacturer’s instructions. The prey vector pGADT7 and bait vector pGBKT7 harboring the genes to be tested were cotransformed into the yeast (*Saccharomyces cerevisiae*) strain AH109. The yeast transformants were spread on SD/-Trp/-Leu agar plates and incubated at 30°C for 2–3 d. Colonies grown on SD/-Trp/-Leu agar plates were suspended in SD/-Trp/-Leu liquid medium to an optical density of 0.6 at 600 nm (OD_600_). A 10× dilution series of 5 µL aliquots of yeast transformants were spotted onto SD/-Trp/-Leu or SD/-Trp/-Leu/-His/-Ade agar plates supplemented with 5-bromo-4-chloro-3-indolyl-α-D-galactopyranoside (X-α-Gal). Then the plates were incubated at 30 °C for 2–3 d. The yeast AH109 cotransformed with pGADT7-T and pGBKT7-53, which interact in Y2H assays, were used as positive controls, and the yeast cotransformed with pGADT7-T and pGBKT7-Lam, which do not interact, were used as negative controls.

For the BiFC assays, two YFP fusion constructs were cotransformed into the *Agrobacterium tumefaciens* GV3101, then cultured to 0.6 at OD_600_. Equal volumes of each culture were mixed and infiltrated into the epidermal cells of *N. benthamiana* leaves using a needleless syringe. The agroinfiltrated plants were cultured under normal growth conditions for 48 to 72 h.

For Co-IP assays, total proteins were extracted as previously described (Vijayapalani et al., 2012) and then centrifuged at 16,000 × *g* for 10 min at 4°C for three times. The supernatants were incubated with anti-mCherry Nanobody Magarose Beads (Shenzhen KangTi Life Technology, Shenzhen, China) overnight at 4°C with gentle shaking. Then the Magarose beads were centrifuged at 500 × *g* for 3 min at 4°C and washed five times with IP buffer. Western blotting was conducted by using mCherry antibodies as described above.

### 2.12 Transient expression

The constructs harboring the genes to be tested were individually transformed into *Agrobacterium*, which were then agroinfiltrated into *N. benthamiana* leaves using needleless syringes. The GV3101 transformants were cultured overnight in Luria–Bertani medium supplemented with the appropriate antibiotics and collected by centrifugation, and were resuspended in 10 mM MgCl_2_ supplemented with 100 mM acetosyringone (Sigma, St. Louis, MO, USA). After incubation at room temperature for 2-3 h, the culture was diluted to an OD_600_ of 0.2-0.5 and agroinfiltrated into the leaves of *N. benthamiana* plants. The agroinfiltrated plants were maintained under normal growth conditions for 48 to 72 h.

### 2.13 Confocal microscopy

Leica SP8 X inverted confocal microscope with an argon laser (Leica, Wetzlar, Germany) was used to image the agroinfiltrated leaf sections at room temperature. YFP was excited at 514 nm, and the emission was captured at 530–590 nm. The RFP was excited at 561 nm, and the emission was captured at 588–648 nm. Images were captured digitally and processed using Leica Application Suite Advanced Fluorescence Lite software (Leica Microsystems). ImageJ (http://rsbweb.nih.gov/ij/) was used to quantify the fluorescence intensity.

## 3 Results

### 3.1 Identification, phylogenetic analysis, and gene structure analysis of the *SsREM* gene family

**Figure 1:**
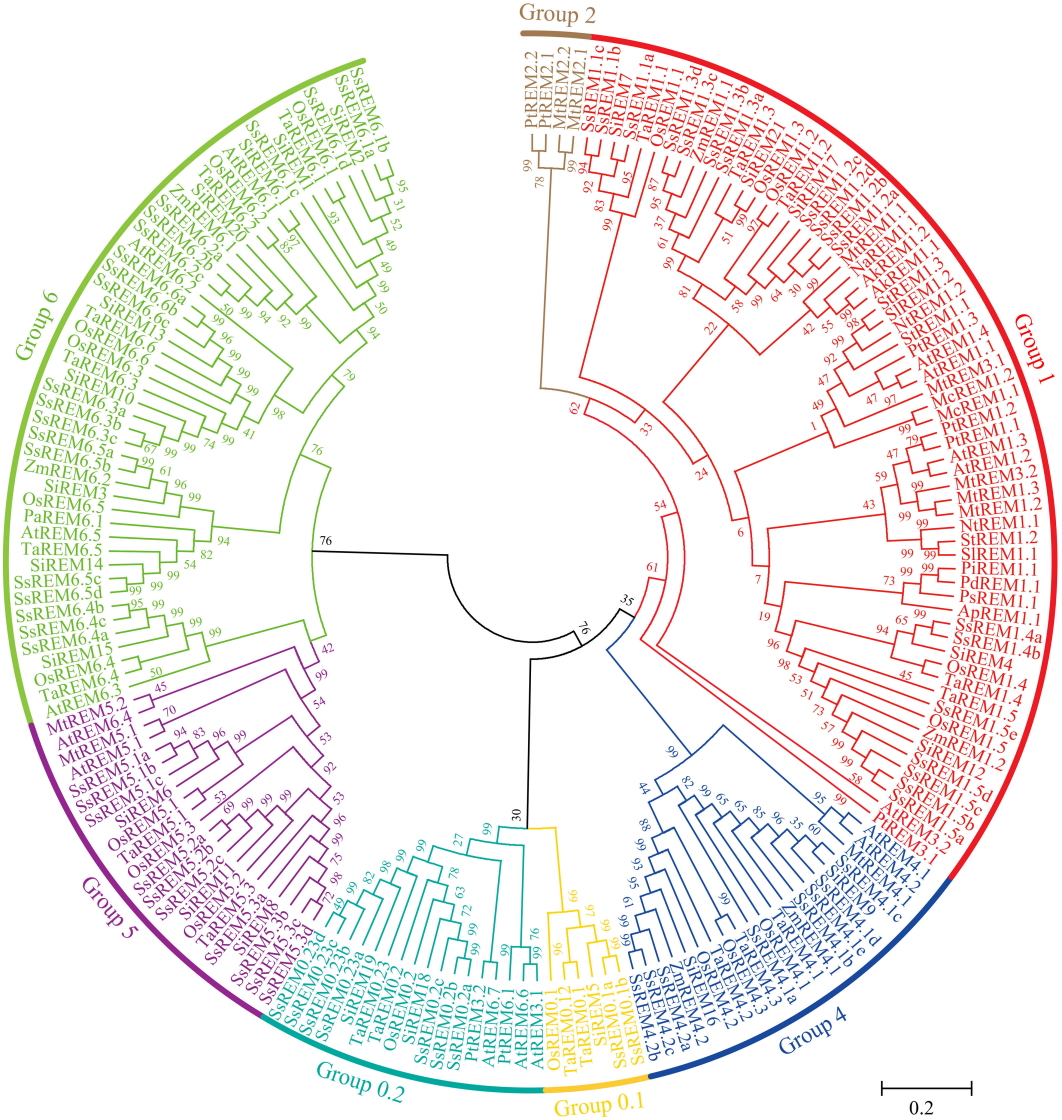
Phylogenetic tree analysis of Remorins (REMs) proteins. The phylogenetic tress was constructed using the maximum likelihood method with 1000 bootstrap replicates. The REMs sequences are from the databases of 5 monocotyledons (*Oryza sativa*, *Zea mays*, *Triticum aestivum*, *Setaria italic*, *Allium cepa*, and *Saccharum spontaneum*), 10 dicotyledons (*Arabidopsis thaliana*, *Solanum tuberosum*, *Solanum lycopersicon*, *Populus trichocarpa*, *Persea Americana*, *Nicotiana tabacum*, *Nuphar advena*, *Medicago truncatula*, *Mesembryanthemum crystallinum* and *Amborella trichopoda*) and 3 gymnosperm plants (*Pinus tadea*, *Pinus pinaster* and *Picea sitchensis*). The *S. spontaneum* remorins were grouped into six distinct groups and annotated with different colors.

A total of 65 *SsREM* genes were identified in the *S. spontaneum* genome (Zhang et al., 2018) based on the N-terminal (Pfam ID PF03766) and C-terminal (Pfam ID PF03763) regions of the REM proteins. To investigate the evolutionary relationships of the *REM* gene family, a phylogenetic tree was constructed to associate the SsREMs with those of five other monocotyledonous plants (*Zea mays*, *Setaria italica*, *Triticum aestivum*, *Oryza sativa*, and *Allium cepa*), ten dicotyledonous plants (*Arabidopsis thaliana*, *Solanum tuberosum*, *Solanum lycopersicum*, *Populus trichocarpa*, *Persea Americana*, *Nicotiana tabacum*, *Nuphar advena*, *Medicago truncatula*, *Mesembryanthemum crystallinum* and *Amborella trichopoda*) and three gymnosperms (*Pinus taeda*, *Pinus pinaster* and *Picea sitchensis*) (Raffaele et al., 2007; Badawi et al., 2019; Wang et al., 2022) (**Supplementary Table 2**). The 65 SsREM proteins were classified into 6 groups (**Figure 1**), namely, Group 0.1, Group 0.2, Group 1, Group 4, Group 5, and Group 6, which contained 2, 7, 18, 8, 10, and 20 members, respectively (**Figure 1**; **Supplementary Figure 1A**; **Supplementary Table 2**). These results are similar to those of previous reports (Raffaele et al., 2007; Badawi et al., 2019; Wang et al., 2022).

The online software MEME (http://meme-suite.org/tools/meme) was used to investigate the motifs contained in the SsREM proteins, and a total of 20 conserved motifs were identified (**Supplementary Figure 1B**). With the exceptions of SsREM1.1c, SsREM1.3a, SsREM1.3b, SsREM4.1a, SsREM5.3a/b/c/d, SsREM6.4a/b/c and SsREM6.5c/d, the SsREM proteins contain Motif 1, Motif 3 and Motif 7; Group 0.1 and Group 5 do not contain Motif 2. Moreover, SsREM0.1a, SsREM1.1b/c, SsREM1.3a/b, and SsREM5.3a contain only three motifs, and Group 0.2, Group 5 and Group 6 only contain Motif 8, Motif 9, Motif 15 and Motif 19. SsREM0.1a and SsREM1.1b contain Motif 1, Motif 3 and Motif 7. SsREM1.1c and SsREM1.3a/b contain Motif 1, Motif 2 and Motif 7. SsREM5.3a contains Motif 1, Motif 4 and Motif 6.

The exon−intron distribution patterns of the *SsREM* genes were also investigated via TBtools (**Supplementary Figure 1C**). The results showed that the number of exons in the *SsREM* genes varied from 1 to 9, and the exons also varied within the same group. For example, in Group 1, SsREM1.2a had only 1 exon, while SsREM1.1a had 6 exons. Most of the genes contained 4 to 6 exons, which is similar to the findings of previous reports (Badawi et al., 2019).

### 3.2 Analysis of *SsREM* gene synteny, duplication, selection pressure, chromosomal location and physicochemical properties

**Figure 2:**
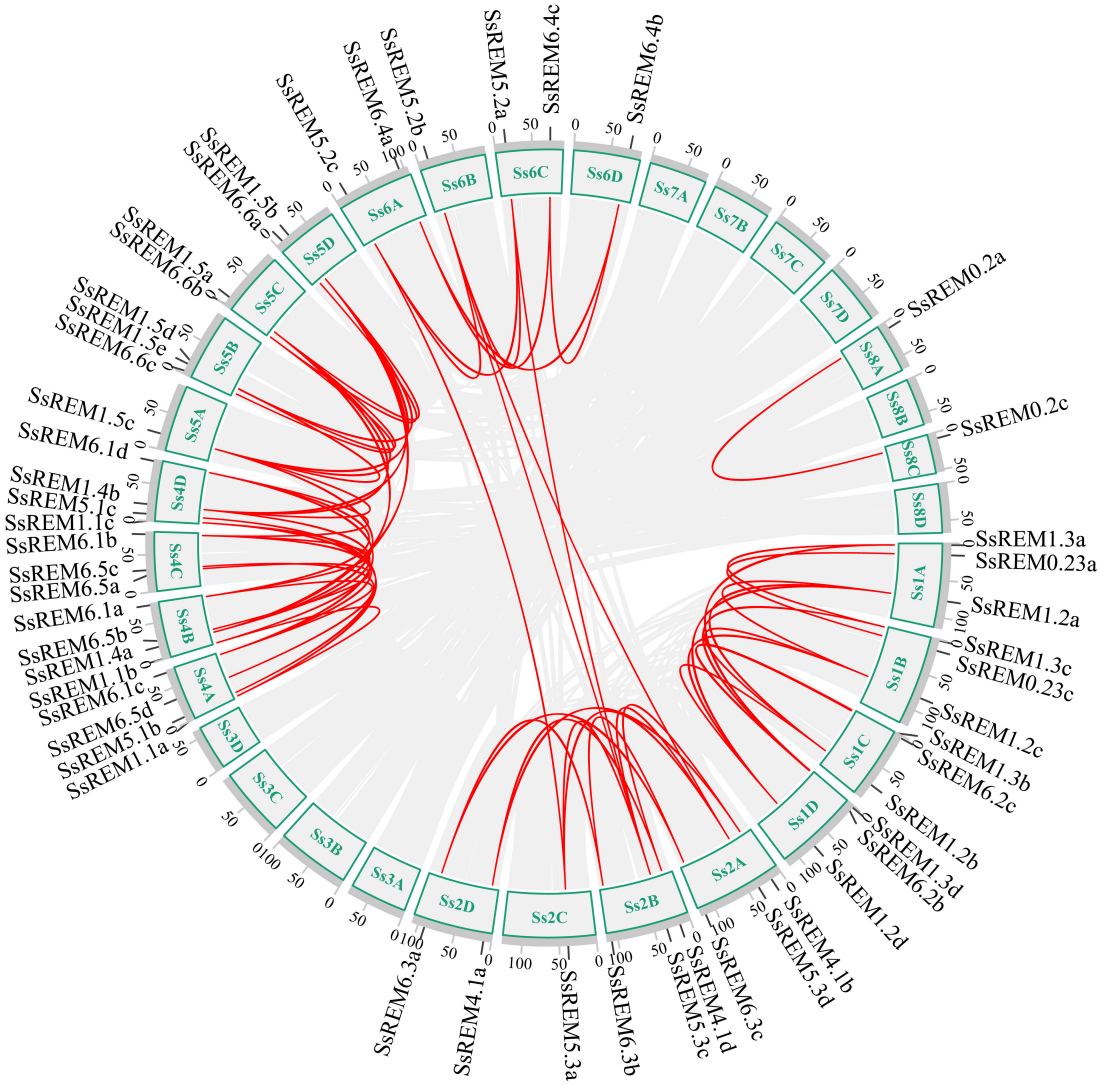
Interchromosomal collinearity relationship analysis of the *REM* gene family from *Saccharum spontaneum*. The red lines represent replicated *REM* gene pairs.

**Figure 3:**
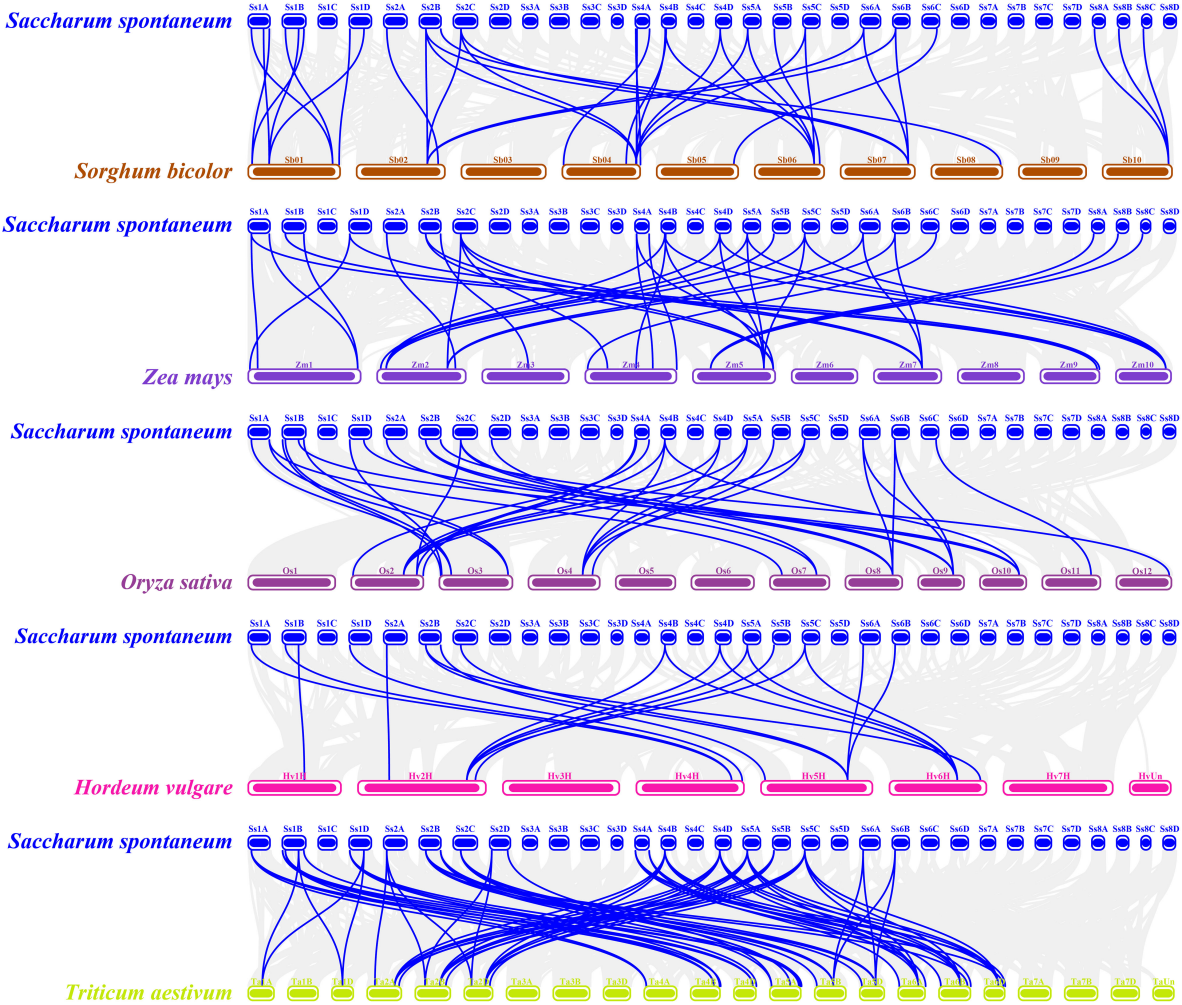
Synteny analysis of *REM* genes between *Saccharum spontaneum* and other five *Poaceae* species. The gray lines in the background represent collinear blocks in the genomes of millet and the other plant species, i.e., *Sorghum bicolor*, *Zea mays*, *Oryza sativa*, *Hordeum vulgare* and *Triticum aestivum*, respectively. The blue lines represent the *SsREM* homologous gene pairs.

To investigate the evolutionary mechanisms of the *SsREM* gene family, the synteny of the *SsREM* gene family within *S. spontaneum* itself was analyzed. As shown in **Figure 2**, there were 60 pairs of homologous *SsREM* genes in the genome of *S. spontaneum*. There were 2, 27, 3, 11 and 17 pairs of homologous *SsREM* genes in Group 0.2, Group 1, Group 4, Group 5 and Group 6, respectively. However, Group 0.1 contained no gene pairs. To obtain additional information on the evolution of the *REM* gene family, synteny analysis was conducted on *S. spontaneum* compared with sorghum, maize, rice, barley, and wheat. The results revealed 37 pairs of homologous genes between *S. spontaneum* and sorghum, 40 pairs between *S. spontaneum* and maize, 33 pairs between *S. spontaneum* and rice, 15 pairs between *S. spontaneum* and barley, and 69 pairs between *S. spontaneum* and wheat (**Figure 3**; **Supplementary Table 3**).

The gene duplication types or events were identified by MCScanX software. As shown in **Supplementary Table 4**, dispersed, tandem and whole-genome duplications (WGD)/segmental duplications were present in the *SsREM* genes. The main duplication types for the *SsREM* genes were WGD/segmental (25/65, 38%) and dispersed (22/65, 34%). The Ka/Ks values of the *SsREM* gene pairs were investigated to evaluate the evolutionary selection pressure on the *SsREM* gene family. As shown in **Supplementary Table 3**, the Ka/Ks ratios of all the gene pairs were < 1, indicating that the homologous genes among *S. spontaneum*, sorghum, maize, rice, barley and wheat had undergone strong purifying selection.

Chromosomal mapping revealed that the *SsREM* genes were unevenly distributed on the 23 chromosomes (**Supplementary Figure 2**; **Supplementary Table 5**), with one on chromosomes 2C, 5A, 6B, 6D, 8A, 8B and 8C (**Supplementary Figure 2**); two on chromosomes 5C, 5D, 6A and 6C (**Supplementary Figure 2**); three on chromosomes 1C, 1D, 2B and 5B (**Supplementary Figure 2**); four on chromosomes 2A, 2D, 4A, 4B and 4C (**Supplementary Figure 2**); and five, six and seven on chromosomes 4D, 1B and 1A, respectively (**Supplementary Figure 2**; **Supplementary Table 5**).

The physicochemical properties and subcellular localization of the 65 SsREM proteins were calculated using the ProtParam tool in ExPASy (**Supplementary Table 6**). The 65 SsREM proteins encoded proteins ranging from 99 to 939 amino acids, with molecular weights ranging from 11.28 kDa to 101.46 kDa. Among them, 53 SsREM proteins were basic proteins, and 12 SsREM proteins were acidic proteins. The instability coefficient indicated that the 65 SsREM proteins were unstable, with the single exception of SsREM4.1b, which was stable. The average hydrophobicity index indicated that all 65 SsREM proteins were hydrophilic. Subcellular localization prediction via Cell-PLoc 2.0 software (online site) revealed that 62 SsREM proteins localize to the plasma membrane, whereas only three SsREM proteins (SsREM6.2a, SsREM6.5b, and SsREM6.6c) localize to the nucleus (**Supplementary Table 6**).

### 3.3 *Cis*-element analysis of the *SsREM* gene family

**Figure 4:**
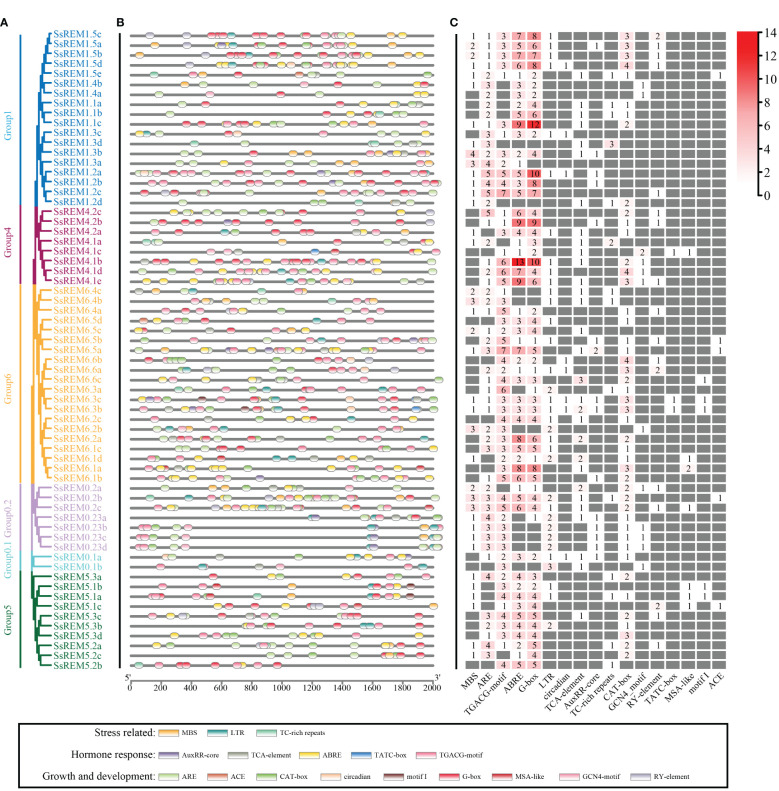
*Cis*-elements analysis of the *REM* gene family in *Saccharum spontaneum*. **(A)** The phylogenetic tree of *SsREM* from *S. spontaneum*. **(B)** Distribution of *cis*-elements in the promoter regions of *SsREM* genes family. **(C)** Number of *cis*-elements in the promoter regions of *SsREM* genes family. Different colored boxes represent different *cis*-elements.

According to the genome of *S. spontaneum*, the *cis*-regulatory elements within the first 2,000 bp upstream of 65 *SsREMs* were predicted. A total of 17 *cis*-regulatory elements were screened; these elements are involved in the responses to stress, growth and development, and hormones (**Figure 4** and **Supplementary Table 7**). More than 80% of the *SsREM* genes contained elements related to anaerobic induction (ARE, 81.5%), methyl jasmonate (TGACG and CGTCA motifs, 86.2%), ABA (ABRE, 83.1%) and light responsiveness (G-box, 84.6%). Among the elements involved in growth and development, the ACE element is present in *SsREM1.5e*, *SsREM5.1e* and *SsREM6.5a/b*; the motif I element is present in *SsREM5.1a/b*, *SsREM6.3b/c* and *SsREM6.6c*, *SsREM0.2c*; and the cell cycle regulation element (MSA-like) is present in *SsREM4.1c*, *SsREM5.1a/b/c* and *SsREM6.1a/d*. Moreover, only *SsREM4.1c* and *SsREM6.3b/c* contain the TATC box. However, *SsREM0.1b*, *SsREM1.3d*, *SsREM1.4a* and *SsREM5.2b* contain 4 *cis*-elements (*SsREM0.1b*: TGACG-motif, LTR, TCA-element and GCN4-motif; *SsREM1.3d*: MBS, ARE, TCA-element and TC-rich repeats; *SsREM1.4a*: ARE, ABRE, G-box and GCN4-motif; *SsREM5.2b*: TGACG-motif, ABRE, G-box and TC-rich repeats) (**Figure 4**; **Supplementary Table 7**).

### 3.4 Expression patterns of *SsREM* genes in sugarcane

**Figure 5:**
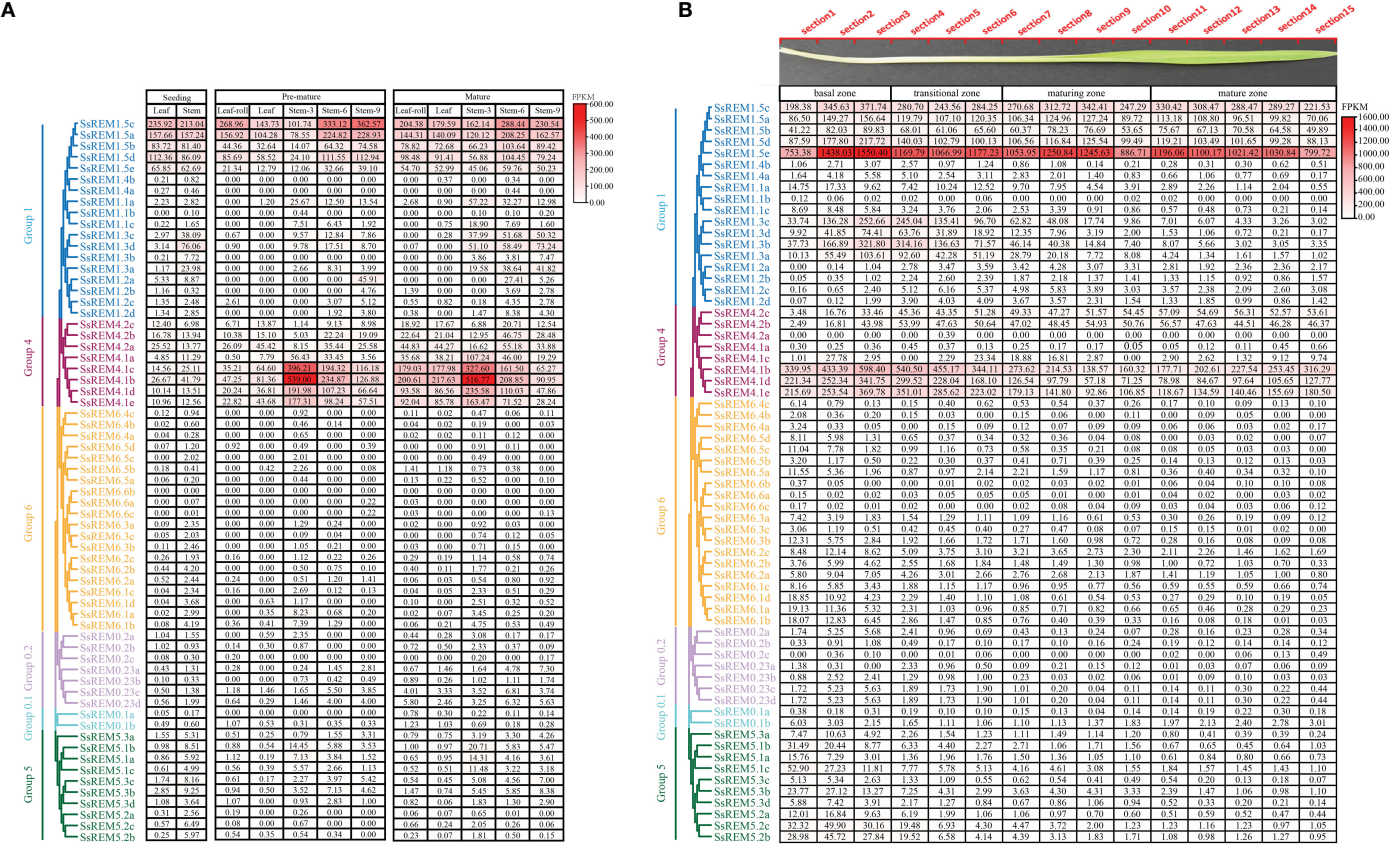
The expression patterns of *Remorin* (*SsREM*) genes family in different tissues and across leaf gradients. **(A)** The expression patterns of *SsREM* gene family based on FPKM in different tissues of different stages in *Saccharum spontaneum*. **(B)** The expression patterns of *SsREM* genes based on FPKM across leaf gradients in *Saccharum spontaneum*.

Transcriptomic data were used to explore the expression patterns of *SsREM* genes in different tissues and developmental stages of *S. spontaneum* (Li et al., 2021). The expression levels of the *SsREM* genes in Group 1 and Group 4 were higher than those in Group 0.1, Group 0.2, Group 5 and Group 6. Moreover, the *SsREM* genes of Group 0.1, Group 0.2, Group 5 and Group 6 were expressed at very low levels or were undetectable in the premature stage and mature stage (**Figure 5A**). Notably, the *SsREM* genes in Group 1 (*SsREM1.5ea/b/c/d/e*) and Group 4 (*SsREM4.1a/b/c/d/e*) were expressed at very high levels in the stems during the premature or mature stage (**Figure 5A**).

To further investigate the functions of the *SsREM* genes in the photosynthetic tissues of *S. spontaneum*, we analyzed the transcriptomes of the leaves along a continuous developmental gradient as described in previous studies (Li et al., 2021). The results showed that the expression levels of the *SsREM* genes were very high in Group 1 and Group 4 but very low in the other groups. Five genes (*SsREM1.5c/e* and *SsREM4.1b/d/e*) exhibited very high expression levels, suggesting that these genes play a key role in leaf development in *S. spontaneum*. *SsREM1.5e* presented the highest expression levels across the 15 sections of the leaf, whereas *SsREM4.2a* exhibited very low or undetectable expression levels (**Figure 5B**). The results indicate that the *SsREM* genes may function differently at various developmental stages, thereby affecting biological processes in different tissues.

### 3.5 Cloning of the *ScREM1.5e-1/-2* genes

**Figure 6:**
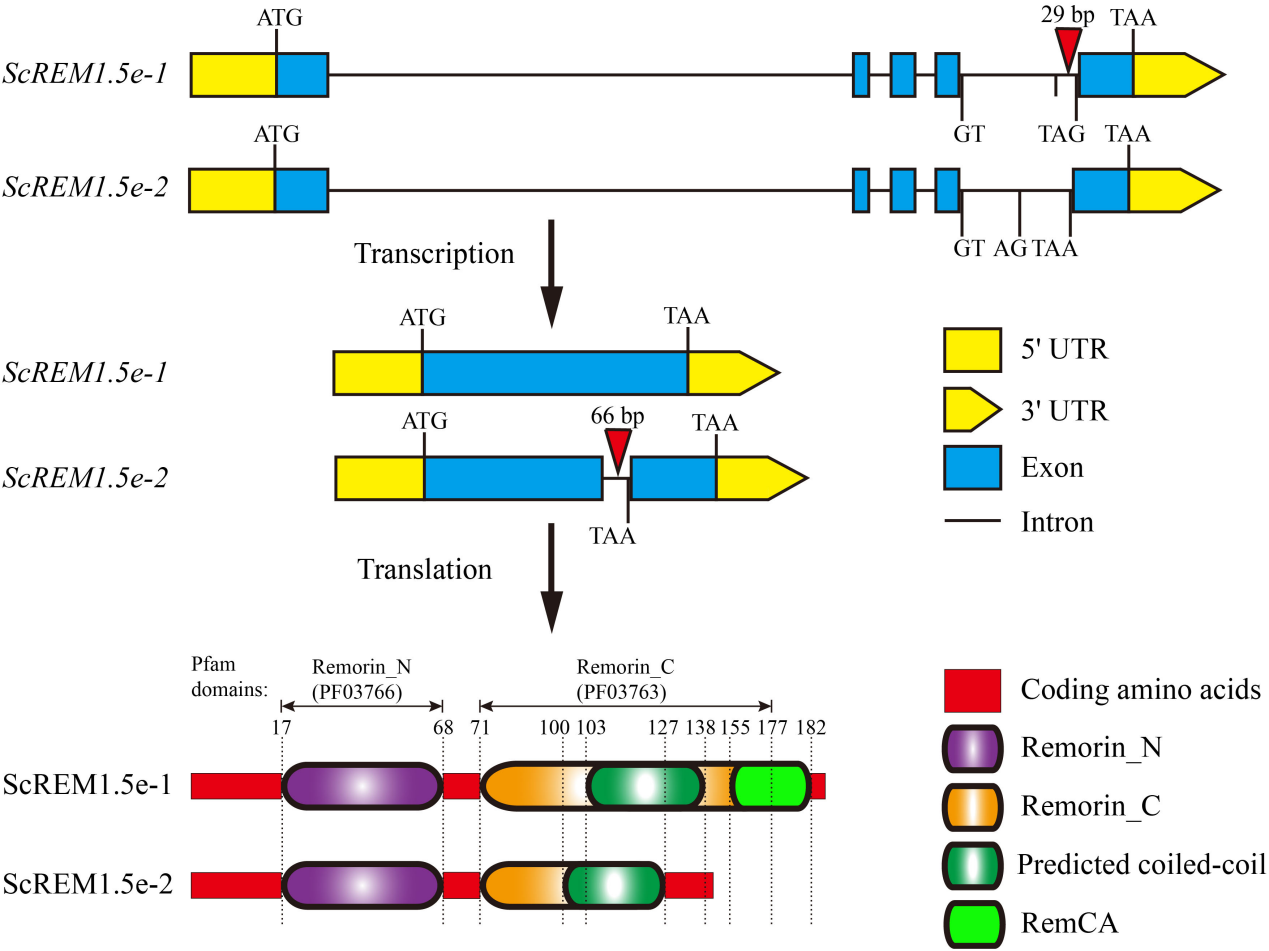
The diagram schematics of gene and protein structures of ScREM1.5e-1 and ScREM1.5e-2. The inverted triangle indicates the insertion of nucleic acids.

Two Group 1 *REM* alleles were cloned from the sugarcane cultivar ROC22 and designated *ScREM1.5e-1* and *ScREM1.5e-2*, respectively, based on phylogenetic tree analysis with the 65 *SsREM* genes (**Supplementary Figure 3**). The open reading frame (ORF) of *ScREM1.5e-1* (OR805348) is 555 bp in length and encodes 184 amino acids. While, the ORF of *ScREM1.5e-2* (OR805349) is 420 bp in length and encodes 139 amino acids. The genomic structures of *ScREM1.5e-1* or *ScREM1.5e-2* are similar to that of Group I *REM* in other plants, with five exons and four introns (**Figure 6**). The difference between *ScREM1.5e-1* or *ScREM1.5e-2* at the genomic level is that the fourth intron of *ScREM1.5e-1* is 29 bp longer than that of *ScREM1.5e-2* (**Figure 6**). The fourth intron of *ScREM1.5e-1* is 239 bp in length with the first 2 nucleic acids at the 5´ terminus are GT and the last 3 nucleic acids at the 3´ terminus are TAG. As GT and AG are conserved splicing sites, the fourth intron of *ScREM1.5e-1* could be successfully be spliced, resulting a typical REM protein, ScREM1.5e-1, which contains conserved Remorin_N domain (PF03766, 17-68), Remorin_C domain (PF03763, 71-177) domain (in which contains a coiled-coil domain) (**Figure 6**). A conserved C-terminal anchor (RemCA) was also found at the C-terminal (155-182) of ScREM1.5e-1 by sequence alignment with the well-studied StREM1.3 or AtREM1.4 (Perraki et al., 2012; Raffaele et al., 2013) (**Figure 6**). However, for the *ScREM1.5e-2*, the splicing sites AG at the end of the fourth intron mutated to AA, resulting failure in splicing. Whereas, the splicing occurred at the A_143_G_144_ of the fourth intron, resulting in retainment of the 66 bp of the fourth intron in the ORF of *ScREM1.5e-2* and a meaningful termination codon TAA (**Figure 6**). Thus, ScREM1.5e-2 protein losses whole RemCA and partial Remorin_C or coiled-coil domain (**Figure 6**).

### 3.6 Expression patterns of *ScREM1.5e-1/-2* in different tissues or upon stress

**Figure 7:**
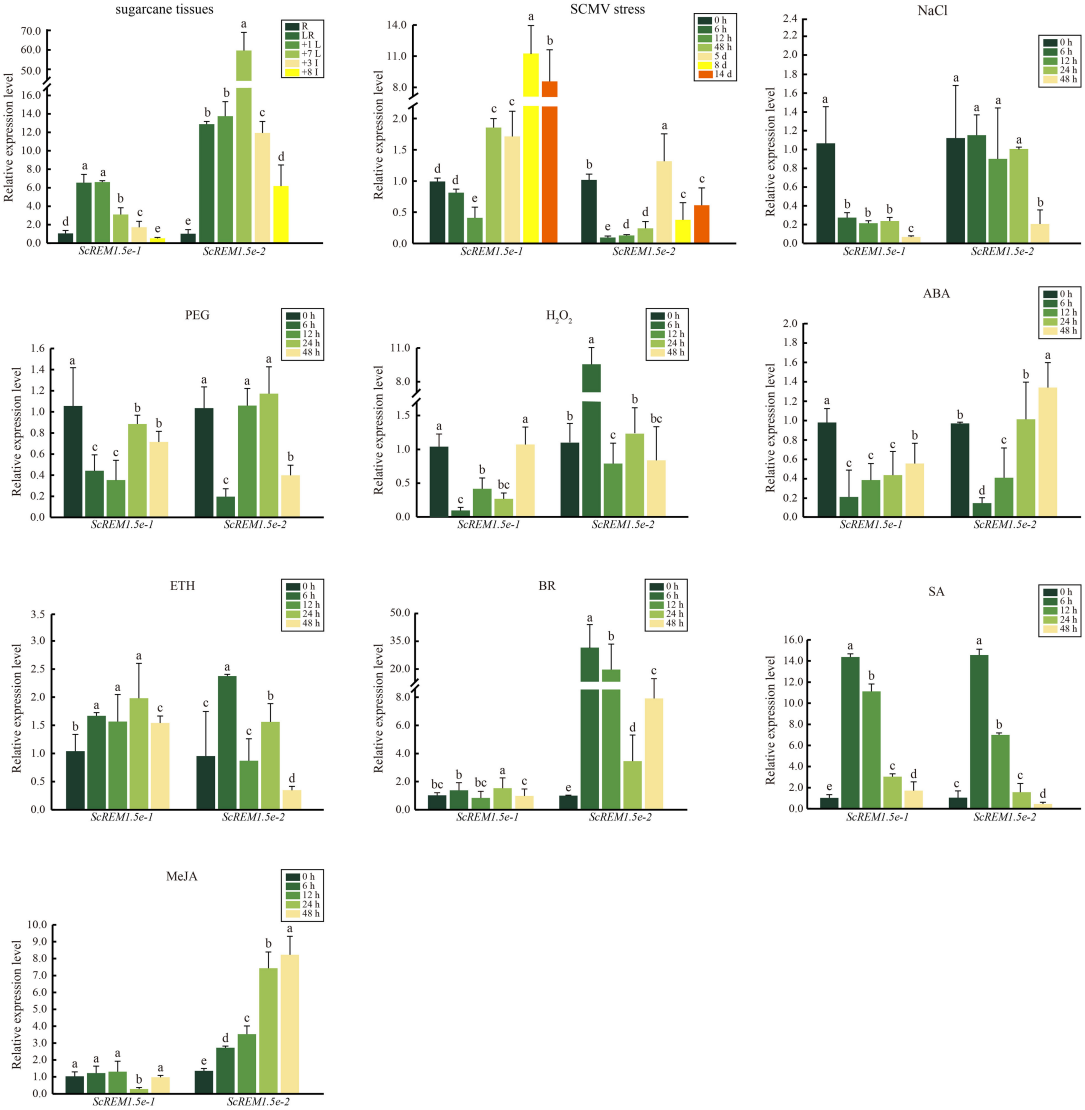
The expression profiles of *ScREM1.5e-1* and *ScREM1.5e-2* in different tissues of sugarcane cultivar ROC22, or under the challenge by *Sugarcane mosaic virus* (SCMV) or other treatments. For the study of the expression patterns of *ScREM1.5e-1* and *ScREM1.5e-2*, the sugarcane cultivar *ROC22* plants about 120-day-old were used. LR: leaf roll; +1 L: 1^st^ leaf; +7 L: 7^st^ leaf; +3 I: the 3^rd^ internode; +8 I: the 8^th^ internode and R: root. For the expression profiles of *ScREM1.5e-1* and *ScREM1.5e-2* genes under the challenge by SCMV, leaves of ROC22 plantlets were inoculated with SCMV and sampled at different time points. Plants mock inoculated with 0.01 mM phosphate buffer (pH 7.0) were used as the negative controls. The Y axes indicates the relative expression of *ScREM1.5e-1/-2*. The X axes indicates the time point of material collection. Error bars indicate SD (*n* = 3), a, b, c, d and e indicate significance at the corresponding time points, Student’s *t*-test, *P* < 0.05. Results were representative of three independent experiments. Simulated plant stresses conditions, including 250 mM NaCl, 25% PEG, 10 mM H_2_O_2_, 100 µM ABA, 400 mg/L ETH, 25 mg/L BR, 5 mM SA or 25 µM MeJA, for 0 h, 12 h, 24 h and 48 h. Error bars indicate SD (*n* = 3), a, b, c, d and e indicate significance at the corresponding time points, Student’s *t*-test, *P* < 0.05. Results were representative of three independent experiments.

The expression patterns of *ScREM1.5e-1/-2* in different tissues of the sugarcane cultivar ROC22 were determined via RT−qPCR analysis. Low expression levels of *ScREM1.5e-1* were observed in the root and 8^th^ internode. However, a greater expression level (> 6-fold greater than that in the roots) of *ScREM1.5e-1* was found in the leaf rolls and 1^st^ leaves than in the 8^th^ internode and roots (**Figure 7**). In addition, the 8^th^ internode presented the lowest *ScREM1.5e-1* expression level (**Figure 7**). However, the lowest expression level of *ScREM1.5e-2* was found in the roots, and the expression levels were significantly greater in the leaves (> 10-fold higher than those in the roots) and internodes (**Figure 7**). These results suggest that *ScREM1.5e-1/-2* may have different functions in the same tissues.

As the Group 1 *remorin* are extensively involved in the response to pathogen infection, we investigated the transcription profiles of *ScREM1.5e-1/-2* in ROC22 plantlets challenged by SCMV using RT−qPCR. The results showed that the expression levels of *ScREM1.5e-1* were significantly downregulated 12 h after SCMV inoculation and subsequently upregulated more than 8-fold at 8 or 14 d (**Figure 7**). However, the expression of *ScREM1.5e-2* was significantly downregulated at 12 h after SCMV inoculation, then significantly (but not more than 1.5-fold) upregulated at 5 d, then downregulated to levels significantly lower than those in the controls (**Figure 7**).

As many *cis*-elements involved in plant responses to stress and hormones were found in the promoters of Group 1 *remorin*, we investigated the expression patterns of *ScREM1.5e-1/-2* in the sugarcane cultivar *ROC22* under various abiotic stresses and hormonal treatments by RT−qPCR. Generally, the expression patterns of *ScREM1.5e-1/-2* were similar under treatment with 25% PEG, 100 µM ABA or 5 mM SA but were different under treatment with 250 mM NaCl, 10 mM H_2_O_2_, 400 mg/L ETH, 25 mg/L BR or 25 µM MeJA (**Figure 7**). In addition, *ScREM1.5e-1/-2* was downregulated at 6 h and then upregulated to the level of the control or slightly lower than the control level under 25% PEG and 100 µM ABA. However, under 5 mM SA, the expression levels of *ScREM1.5e-1/-2* at 6 h and 12 h were 6- to14-fold greater than those in the controls. 250 mM NaCl treatment significantly suppressed the expression of *ScREM1.5e-1* but had no impact on the expression of *ScREM1.5e-2* except at 48 h. Treatment with 10 mM H_2_O_2_ also significantly suppressed the expression of *ScREM1.5e-1* but significantly promoted the expression of *ScREM1.5e-2* at 6 h. In particular, 25 mg/L BR or 25 µM MeJA did not impact the expression levels of *ScREM1.5e-1* but significantly upregulated the expression of *ScREM1.5e-2*. In the 400 mg/L ETH treatment, *ScREM1.5e-1* had a greater expression level than the control at 6 h, 12 h, 24 h, and 48 h. Inversely, *ScREM1.5e-1* had a higher expression level than the control only at 6 h.

### 3.7 Interaction of ScREM1.5e-1/-2 with SCMV-/SCSMV-/SrMV-VPg

In our previous study, we found that the VPg of TuMV interacts with AtREM1.2/1.3 to facilitate virus infection, and this interaction relocalizes AtREM1.2/1.3 from the plasma membrane to the cytoplasm (Cheng et al., 2020). To test the possible influence of SCMV-/SCSMV-/SrMV-VPg on the subcellular localization of ScREM1.5e-1/-2, we first investigated the subcellular localization of ScREM1.5e-1/-2 or SCMV-/SCSMV-/SrMV-VPg in the epidermal cells of *N. benthamiana* leaves. The recombinant plasmids YFP-ScREM1.5e-1/-2 and mCherry-AtREM1.2, HDEL-RFP or H2B-RFP were subsequently transformed into *N. benthamiana* leaves by agroinfiltration as previously described (Cheng et al., 2020). Confocal observation was conducted at 48 hpi. The YFP-ScREM1.5e-1 fluorescence was strongly distributed on the plasma membrane and merged with the red fluorescence of RFP-AtREM1.2, a PM marker, indicating the PM localization of ScREM1.5e-1 (**Supplementary Figure 4A**). However, the fluorescent signal of YFP-ScREM1.5e-2 merged with the fluorescent signal of HDEL-RFP, a cytoplasmic marker, or H2B-RFP, a nuclear marker (**Supplementary Figure 4A**), indicating the cytoplasmic or nuclear localization of ScREM1.5e-2. For SCMV-/SCSMV-/SrMV-VPg-YFP, the fluorescent signals merged with those of HDEL-RFP or H2B-RFP, indicating the cytoplasmic and nuclear localization of SCMV-/SCSMV-/SrMV-VP, respectively (**Supplementary Figures 4B, C**). All the localization assay results were further confirmed by fluorescence intensity measurements (**Supplementary Figure 4**).

**Figure 8:**
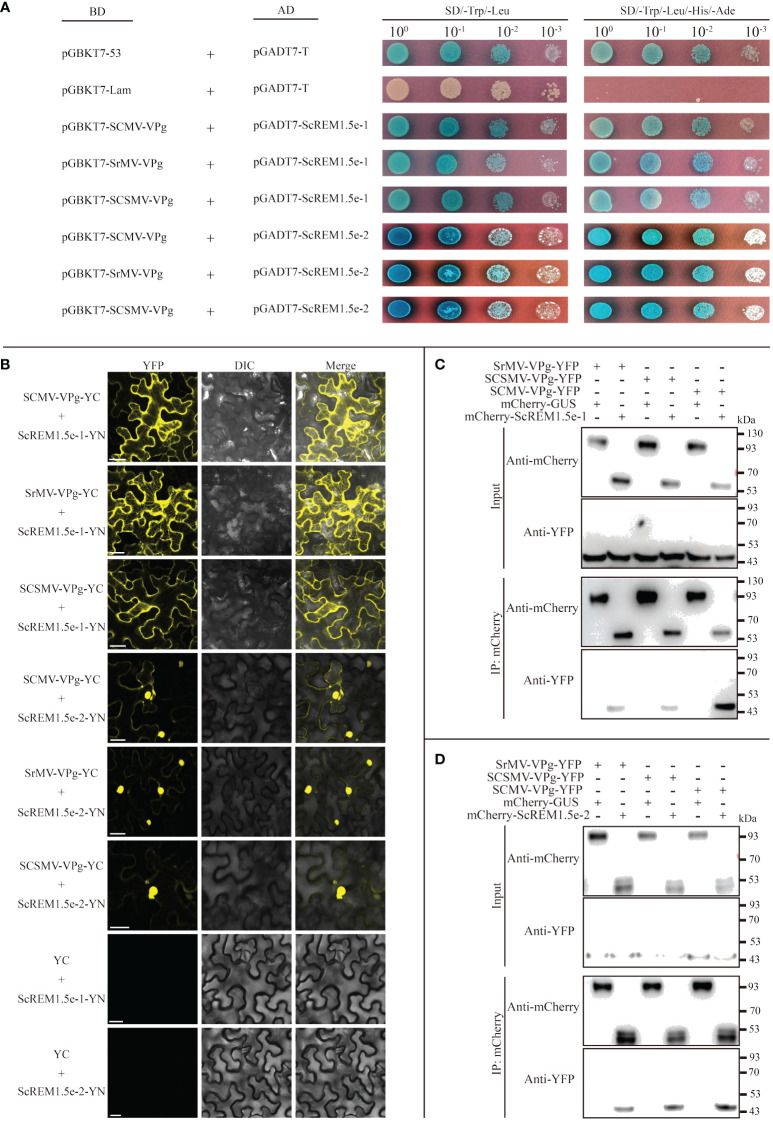
Interaction of ScREM1.5e-1or ScREM1.5e-2 with SCMV-/SCSMV-/SrMV-VPg. **(A)** Y2H assays. pGADT7-ScREM1.5e-1 or pGADT7-ScREM1.5e-2 were individually pairwise co-transformed with the vector pGBKT7-SCMV-VPg, pGBKT7-SCSMV-VPg, or pGBKT7-SrMV-VPg into the yeast AH109 cells in a 10 × dilution series of 10-µL aliquots, which were then plated on a non-selective medium SD/-Leu/-Trp or quadruple dropout medium SD/-Leu/-Trp/-His/-Ade supplemented with X-α-Gal. Yeast cells co-transformed with pGBKT7-53 and pGADT7-T were used as a positive control, pGBKT7-Lam and pGADT7-T were used as a negative control. **(B)** BiFC assays. Agrobacteria harboring YC/YN fusion proteins were individually pairwise co-infiltrated into *N. benthamiana* leaves. The leaf epidermal cells pairwise co-transformed with ScREM1.5e-1-YN or ScREM1.5e-12-YN plus YC were used as negative controls. The images were captured at 48 h post infiltration. Bars = 25 µm. **(C, D)** Co-immunoprecipitation (co-IP) assays to confirm the interaction of ScREM1.5e-1 or ScREM1.5e-2 with SCMV-/SCSMV-/SrMV-VPg, respectively. Total proteins were extracted from *N. benthamiana* leaves expressing the mCherry-ScREM1.5e-1/-2, mCherry-GUS or SCMV-/SCSMV-/SrMV-VPg-YFP construct. The immune complexes were immobilized on anti-mCherry magnetic beads, and the co-precipitation of SCMV-/SCSMV-/SrMV-VPg were examined by western blotting using antibodies against YFP or mCherry.

To test the interaction of ScREM1.5e-1/-2 with SCMV, SCSMV, and SrMV-VPg, Y2H assays based on GAL4 and BiFC assays were conducted. For the Y2H assays, pGBKT7-SCMV-/SCSMV-/SrMV-VPg was individually cotransformed with pGADT7-ScREM1.5e-1/-2 into the yeast strain AH109. The combination of pGBKT7-53 and pGADT7-T was used as a positive control, while the combination of pGBKT7-Lam and pGADT7-T was used as a negative control. The results showed that the yeast cells cotransformed with the combination of pGBKT7-SCMV-/SCSMV-/SrMV-VPg with pGADT7-ScREM1.5e-1/-2 produced blue colonies on SD/-Trp/-Leu and SD/-Trp/-Leu/-His/-Ade culture medium supplemented with X-α-Gal as the positive control (**Figure 8A**), indicating an interaction between SCMV-/SCSMV-/SrMV-VPg and ScREM1.5e-1/-2, while no colonies developed for the negative control (**Figure 8A**).

For the BiFC assays, the fusion construct SCMV-/SCSMV-/SrMV-VPg-YC was individually cotransformed with ScREM1.5e-1/-2-YN into the *Agrobacterium* strain GV3101 and then agroinfiltrated into *N. benthamiana* leaves. Confocal observation was performed at 48 hpi. The results showed that SCMV-VPg, SCSMV-VPg and SrMV-VPg interact with ScREM1.5e-1/-2 individually; SCMV-VPg, SSMV-VPg and SrMV-VPg interact with ScREM1.5e-1 mainly in the cytoplasm, whereas the interaction with ScREM1.5e-2 mainly occurs in the nucleus (**Figure 8B**). As expected, the negative controls ScREM1.5e-1/-2-YN and YC emitted no fluorescence signals (**Figure 8B**).

To further confirm the interaction of SCMV-/SCSMV-/SrMV-VPg with ScREM1.5e-1/-2, Co-IP assays were performed. We found that SCMV-/SCSMV-/SrMV-VPg could be individually pulled down by ScREM1.5e-1/2 but not GUS (**Figures 8C, D**). Collectively, these results demonstrated the interaction of ScREM1.5e-1/-2 with SCMV-/SCSMV-/SrMV-VPg both *in vitro* and *in vivo* (**Figure 8**).

### 3.8 Self-interaction of and interaction between ScREM1.5e-1/-2

**Figure 9:**
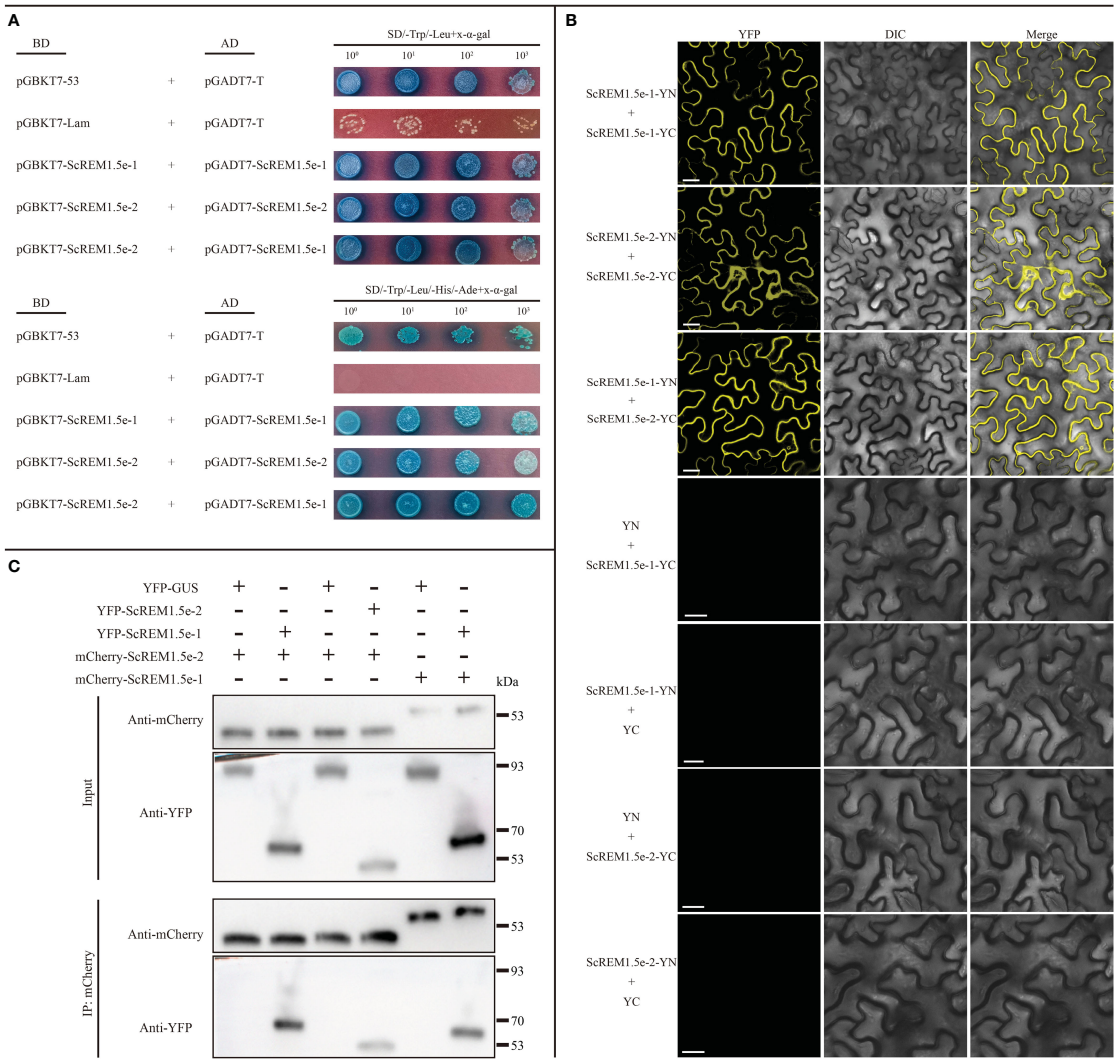
Selfinteraction of or interaction between ScREM1.5e-1 and ScREM1.5e-2. **(A)** Y2H assays. The pGADT7-ScREM1.5e-1 or pGADT7-ScREM1.5e-2 were infused into the prey vector pGADT7 or pGBKT7, then pairwise co-transformed into the yeast AH109 cells in a 10× dilution series of 10-µL aliquots, which were then plated on a non-selective medium SD/-Leu/-Trp or quadruple dropout medium SD/-Leu/-Trp/-His/-Ade supplemented with X-α-Gal. Yeast cells co-transformed with pGBKT7-53 and pGADT7-T were used as a positive control, while the yeast cells co-transformed with pGBKT7-Lam and pGADT7-T were used as a negative control. **(B)** BiFC assays. Agrobacteria harboring YC/YN fusion proteins were co-infiltrated into *N. benthamiana* leaves, respectively. The leaf epidermal cells pairwise co-transformed with YN plus ScREM1.5e-1-YC, ScREM1.5e-1-YN plus YC, YN plus ScREM1.5e-2-YC, or ScREM1.5e-1-YN-plus YC were used as negative controls. The images were captured at 48 h post agroinfiltration. Bars = 25 µm. **(C)** Co-IP assays to confirm the interaction of ScREM1.5e-1 with ScREM1.5e-2 or the self-interaction of ScREM1.5e-1 or ScREM1.5e-2. Total proteins were extracted from *N. benthamiana* leaves individually expressing the YFP-ScREM1.5e-1, YFP-ScREM1.5e-2, YFP-GUS, mCherry-ScREM1.5e-1, or mCherry-ScREM1.5e-2 construct. The immune complexes were immobilized on anti-mCherry magnetic beads, and the co-precipitation of ScREM1.5e-1 or ScREM1.5e-2 were examined by western blotting using antibodies against YFP or mCherry.

As previous studies have shown that REMs can form homo or hetero-oligomers (Keinath et al., 2010; Martinez et al., 2019; Cai et al., 2020; Ma et al., 2022; Su et al., 2023), we performed Y2H, BiFC and Co-IP assays to evaluate the self-interaction of ScREM1.5e-1/-2 or the interaction between ScREM1.5e-1 and ScREM1.5e-2. For the Y2H assays, the combinations of pGBKT7-ScREM1.5e-1 plus pGADT7-ScREM1.5e-1, pGBKT7-ScREM1.5e-1 plus pGADT7-ScREM1.5e-2, and pGBKT7-ScREM1.5e-2 plus pGADT7-ScREM1.5e-2 were individually cotransformed into the yeast strain AH109. Like yeast cells cotransformed with the positive control plasmids pGBKT7-53 and pGADT7-T, yeast cells cotransformed with the combination of pGBKT7-ScREM1.5e-1 and pGADT7-ScREM1.5e-1, pGBKT7-ScREM1.5e-1 and pGADT7-ScREM1.5e-2, pGBKT7-ScREM1.5e-2 and pGADT7-ScREM1.5e-2 produced blue colonies on SD/-Trp/-Leu and SD/-Trp/-Leu/-His/-Ade culture medium supplemented with X-α-Gal, indicating interactions between ScREM1.5e-1 and ScREM1.5e-2 and self-interactions (**Figure 9A**), and the negative control cotransformed with plasmids pGBKT7-Lam and pGADT7-T did not produce blue colonies (**Figure 9A**). For the BiFC assays, the fusion construct ScREM1.5e-1/-2-YN/-YC was individually cotransformed with ScREM1.5e-1/-2-YN/-YC into the *Agrobacterium* strain GV3101 and then agroinfiltrated into *N. benthamiana* leaves. At 48 hpi, yellow fluorescence corresponding to YFP was observed via confocal microscopy. The results confirmed the ScREM1.5e-1/-2 interaction and their self-interactions (**Figure 9B**). As expected, the negative controls ScREM1.5e-1/-2-YN/-YC and YC/YN emitted no fluorescence signals (**Figure 9B**).

To further confirm the interaction between ScREM1.5e-1/-2 and the self-interactions, we conducted Co-IP assays. The results showed that ScREM1.5e-1/-2 could be pulled down by ScREM1.5e-1/-2 but not by GUS (**Figure 9C**). Collectively, these results demonstrate the interaction between ScREM1.5e-1 and ScREM1.5e-2 and the self-interactions of each protein both *in vitro* and *in vivo*.

## 4 Discussion

In view of the important role of *REMs* in plant growth and development and in response to biological and abiotic stresses (Raffaele et al., 2007; Jarsch and Ott, 2011; Gouguet et al., 2021), the number of studies on the *REM* gene family at the whole-genome level has gradually increased in recent years (Raffaele et al., 2007; Jamann et al., 2016; Badawi et al., 2019; Wang et al., 2022; Li et al., 2023). In the present study, 65 *REM* genes were identified from *S. spontaneum* (**Figure 1**). There are different numbers of *REMs* in different monocotyledonous crops, e.g., 19 in rice, 20 in wheat, 21 in foxtail millet, 21 in sorghum, and 33 in maize (Raffaele et al., 2007; Badawi et al., 2019; Wang et al., 2022; Li et al., 2023). The numbers of *REMs* are generally comparable across species. For *Panicoideae* crops, the number of *REMs* in foxtail millet is comparable to that in sorghum, while maize has a greater number of *REMs*, which might be due to the tetraploidization of the maize genome 5-12 million years ago (Schnable et al., 2011). However, the number of *REMs* in *S. spontaneum* is 3 times that in sorghum, which is speculated to be related to two whole-genome duplication events in sugarcane (Zhang et al., 2018, 2022). The expansion of gene families is always accompanied by gene duplication. The present study showed that the expansion of the *REM* gene family in *S. spontaneum* occurred mainly through WGD/segmentation and dispersed replication, which is consistent with the findings of previous studies (Wang et al., 2018; Zhang et al., 2018). In the present study, the *S. spontaneum* REMs were classified into 6 groups, in which Group 0.2 may exist only in monocotyledonous plants, while Group 4 may be differentiated in monocots and dicots (**Figure 1**), which is consistent with the findings of previous studies (Raffaele et al., 2007; Badawi et al., 2019).

*REM* gene families are extensively involved in plant growth and development and exhibit diverse expression patterns (Huang et al., 2019; Li et al., 2023). In this study, the transcriptomic data showed that Group 1 REMs (*SsREM1.5a/b/c/d/e*) and Group 4 REMs (*SsREM4.1a/b/c/d/e*) were highly expressed in internodes and leaves (**Figure 5**). However, the expression levels of *ScREM1.5e-1/-2* were significantly greater in leaves than in internodes or roots, with the expression level of *ScREM1.5e-2* being much greater than that of *ScREM1.5e-1* in the sugarcane cultivar ROC22 (**Figure 7**), indicating the different roles of these REM alleles in sugarcane growth and development.

During the growth and development process, crops are subjected to various biotic and abiotic stresses, which seriously affect production and yield (Zhu, 2016; Gong et al., 2020; Ding and Yang, 2022; Waadt et al., 2022). Therefore, crops adopt complex mechanisms to cope with stress, among which transcriptional reprogramming is one of the most important means (Kumar, 2014; Jones, 2016; Carvalhais et al., 2017; Cheng et al., 2018; Xie et al., 2018; Wu et al., 2021; Shang et al., 2023). Transcriptomic studies have shown that *REMs* respond to drought, high salt concentrations, low temperature, ABA, BR, SA and MeJA (Bray, 2002; Reddy et al., 2002; Nohzadeh Malakshah et al., 2007; Checker and Khurana, 2013; Hu et al., 2013; Yue et al., 2014; Gui et al., 2016; Kong et al., 2016; Badawi et al., 2019; Huang et al., 2019; Wang et al., 2022). For example, the abscisic acid (ABA)-responsive DREB-binding protein (SiARDP) from foxtail millet can regulate the expression of *SiREM6* (Li et al., 2014). The tolerance of mutant Arabidopsis plants overexpressing *SiREM6* to high salt and low temperature improved with the exogenous application of ABA (Checker and Khurana, 2013; Li et al., 2014; Yue et al., 2014). ABA activates the transcription factor OsbZIP23 to upregulate the expression of *OsREM4.1* in rice, whereas *OsREM4.1* negatively regulates the BR signaling pathway (Gui et al., 2016). Overexpression of mulberry (*Morus indica*) *MiREM* confers tolerance to drought and salt stresses in mutant Arabidopsis plants (Checker and Khurana, 2013). The expression of the Antarctic hairgrass (*Deschampsia antarctica*) transcription factor DaCBF7 was induced by drought, low temperature, and salinity; however, overexpression of *DaCBF7* in rice plants only conferred cold tolerance (Byun et al., 2015). Further investigation revealed that *REMs* were upregulated, and putative CRT/DRE or low-temperature responsive elements were found in their promoter regions (Byun et al., 2015). Analysis of the *REM* families of Arabidopsis, foxtail millet, rice, wheat and tomato showed that their upstream promoter regions contain a large number of responsive elements related to growth, development, hormones and stress (Raffaele et al., 2007; Yue et al., 2014; Badawi et al., 2019; Wang et al., 2022; Li et al., 2023). Different *REMs* possess different responsive elements in their upstream promoter regions, thereby exhibiting different expression profiles in some situations. For example, most wheat *REMs* respond to ABA induction, but only Group 4 *REMs* are strongly induced under cold stress (Badawi et al., 2019). Our analysis of *cis*-acting elements in the promoter regions of *SsREMs* demonstrated that a large number of elements are involved in the regulation of growth and development and in responses to stress and hormones (**Figure 4**). Therefore, we speculate that the *REMs* of *S. spontaneum* might be widely involved in these biological processes and exhibit different expression patterns, as indicated in **Figure 4**. The upstream promoter regions of *SsREM1.5e* contain MBS, ARE, TGACG-motif, ABRE, G-box, TCA-element, TC-rich repeat, CATbox, and ACE elements (**Figure 4**), resulting in different expression profiles of *ScREM1.5e-1/-2* under different treatments (**Figure 7**). The present study showed that both *ScREM1.5e-1* and *ScREM1.5e-2* responded to PEG or H_2_O_2_ treatment; however, *ScREM1.5e-1* was downregulated under NaCl treatment, whereas *ScREM1.5e-2* was not (**Figure 7**). These results indicate that different REMs might play different roles in response to abiotic stress in sugarcane, and further investigation is needed.

Our previous study showed that TuMV employs P3N-PIPO to recruit PCaP1 to degrade actin filaments in close proximity to or within plasmodesmata to promote TuMV cell-to-cell movement. However, REM interacts with PCaP1 to interfere with the binding of PCaP1 to actin filaments. As a counteractive response, TuMV employs VPg to interact with and mediate REM degradation through autophagy and the 26S proteasome pathway to establish systemic infection (Cheng et al., 2020). Our previous study also confirmed the interaction of SCMV P3N-PIPO with ScPCaP1 (Cheng et al., 2017). In this study, we found that SCMV infection upregulated the expression of *ScREM1.5e-1* and *ScREM1.5e-2*, and the former was significantly greater than the latter (**Figure 7**). In general, SCMV infection significantly upregulated the expression of *ScREM1.5e-1* 2 d post-inoculation but downregulated the expression of *ScREM1.5e-2* (**Figure 7**). It is worth investigating whether ScREM1.5e-1 or ScREM1.5e-2 can interfere with the interaction of ScPCaP1 with actin filaments. The C-terminal anchor RemCA plays key role in the PM localization of REMs (Perraki et al., 2012). In the present study, the subcellular localization assays revealed that ScREM1.5e-1 was localized to the plasma membrane, while ScREM1.5e-2 was localized to the cytoplasm and nucleus (**Supplementary Figure 4**), possibly due to the loss of RemCA (**Figure 6**). Therefore, we speculate that C-terminal anchors are critical for the localization of REM protein plasma membrane. VPg plays an important role in the translation of the potyvirus genome. In addition, ScREM1.5e-1 interacts with the VPgs of SCMV, SCSMV, and SrMV in the plasma membrane and cytoplasm; however, ScREM1.5e-2 interacts with these proteins in the nucleus (**Figure 8B**) and might differentially impact the function of these proteins. In our previous study, we predicted in silico that SCMV, SCSMV, and SrMV-VPg all contain domains that interact with ATG8 (Yang et al., 2020). However, further studies are needed to investigate whether the interaction of ScREM1.5e-1 or ScREM1.5e-2 with all the VPgs interferes with the interaction of SCMV-/SCSMV-/SrMV-VPg with SceIF4Es, thereby impairing the translation of viral genomes or mediating ScREM1.5e-1 or ScREM1.5e-2 degradation via the autophagy pathway or the 26S proteasome pathway. REMs can oligomerize to homologous or heterologous dimers or trimers, thereby participating in the initial immune response of plants (Keinath et al., 2010; Cai et al., 2020; Ma et al., 2022; Su et al., 2023). In the present study, we found that ScREM1.5e-1 and ScREM1.5e-2 self-interact and interact with each other (**Figure 9**), indicating that they can oligomerize. We speculated that oligomerized REMs may also reduce plasma membrane fluidity and plasmodesmata permeability, thereby inhibiting SCMV infection, reminiscent of REMs inhibiting CMV infection (Huang et al., 2019). It would be interesting to further investigate the biological roles of hetero-oligomers of ScREM1.5e-1 with ScREM1.5e-2 because the interaction of ScREM1.5e-1 with ScREM1.5e-2 occurs on the plasma membrane and changes the cytoplasmic localization of ScREM1.5e-2 (**Figure 9B**), suggesting that the biological function of ScREM1.5e-2 is affected to some extent.

## 5 Conclusion

In this study, 65 *REM* genes were identified in *S. spontaneum*. *SsREM* gene duplication events were mainly dispersed and characterized as WGD or segmental. The upstream promoter regions of the *SsREM* family contain multiple *cis*-acting elements associated with stress, growth, and hormonal responses, indicating that the *SsREM* family may be involved in the response to various stresses, growth and development. *SsREMs* were constitutively expressed in different sugarcane tissues, as indicated by the RNA-seq database. In addition, a pair of alleles, *ScREM1.5e-1* and *ScREM1.5e-2*, was cloned from the sugarcane cultivar ROC22. In addition, *ScREM1.5e-1* and *ScREM1.5e-2* were highly expressed in leaves, while the expression of *ScREM1.5e-2* was significantly higher than that of *ScREM1.5e-1* in the internodes. Exogenous application of ABA, ETH, SA or SCMV infection upregulated the expression of *ScREM1.5e-1* and *ScREM1.5e-2*. ScREM1.5e-1 was localized to the PM, while ScREM1.5e-2 was localized to the nucleus and cytoplasm. The interaction of ScREM1.5e-1 or ScREM1.5e-2 with the VPgs of SCMV, SCSMV, or SrMV was individually confirmed by Y2H, BiFC, and co-IP assays. The homo- or hetero-oligomerization of ScREM1.5e-1 with ScREM1.5e-2 was also confirmed. The present study sheds light on the biological functions of ScREM1.5e-1 and ScREM1.5e-2 and will be valuable for engineering sugarcane resistance to sugarcane mosaic disease.

## Data availability statement

The original contributions presented in the study are included in the article/**Supplementary Material**. Further inquiries can be directed to the corresponding authors.

## Author contributions

ZY: Data curation, Writing – original draft, Formal Analysis, Investigation, Visualization. GC: Data curation, Formal Analysis, Investigation, Visualization, Writing – original draft, Funding acquisition. QY: Data curation, Formal Analysis, Investigation, Writing – original draft. KZ: Data curation, Formal Analysis, Investigation, Writing – original draft. WJ: Data curation, Formal Analysis, Investigation, Writing – original draft. TL: Data curation, Formal Analysis, Investigation, Writing – original draft. HZ: Data curation, Formal Analysis, Investigation, Writing – original draft. HS: Data curation, Formal Analysis, Writing – original draft. GH: Writing – original draft, Resources. FW: Resources, Conceptualization, Formal Analysis, Writing – review & editing. YG: Conceptualization, Formal Analysis, Resources, Writing – review & editing, Supervision. JX: Conceptualization, Supervision, Writing – review & editing, Data curation, Funding acquisition, Project administration, Writing – original draft.

## Supplementary material

Supplementary Figure 1 Phylogenetic relationships, conserved motifs and structure analysis of the *Remorin* (*SsREM*) genes family. **(A)** The phylogenetic tree of Remorin proteins in Saccharum spontaneum. **(B)** Conserved motifs analysis of the *Remorin* genes family. **(C)** Gene structure analysis of the Remorin genes family. The groupings of clusters are shown in different colors. Conserved motifs of Remorin proteins are indicated by differently colored boxes. Offwhite boxes represent UTR (untranslated region), dark blue boxes represent exon, black line represent intron.

Supplementary Figure 2 Chromosomal locations of *Remorin* (*SsREM*) genes in the *Saccharum spontaneum* genome. The distributions of the 65 *Remorin* genes were determined according to the scaffold number and are shown in red. The numbers on the top indicate each chromosome number and the genome.

Supplementary Figure 3 Evolutionary tree of the two genes of sugarcane cultivar ROC22 with 65 *SsREM* genes. The two genes of sugarcane cultivar ROC22 were highlighted by the “leaves” in red, indicated that it belongs to REM1.5e branch.

Supplementary Figure 4 Subcellular localization of ScREM1.5e-1/-2 or SCMV-/SCSMV-/SrMV-VPg in the epidermal cells of *Nicotiana benthamiana*. **(A)** Subcellular localization of YFP-tagged ScREM1.5e-1/-2 in *N. benthamiana* leaf epidermal cells. Bars = 20 μm. **(B, C)** Subcellular localization of YFP-tagged SCMV-/SCSMV-/SrMV-VPg in *N. benthamiana* leaf epidermal cells. The fifth column shows overlapping fluorescence spectra analysis of YFP and RFP signals marked in red dashed line. Bars = 20 μm.
