# Supplementary figures and images for: Identification and characterization of the Remorin gene family in Saccharum and the involvement of ScREM1.5e-1/-2 in SCMV infection on sugarcane
Source: Front Plant Sci. 2024 Feb 23;15:1365995. doi: 10.3389/fpls.2024.1365995 (PMC10920289; doi:10.3389/fpls.2024.1365995)

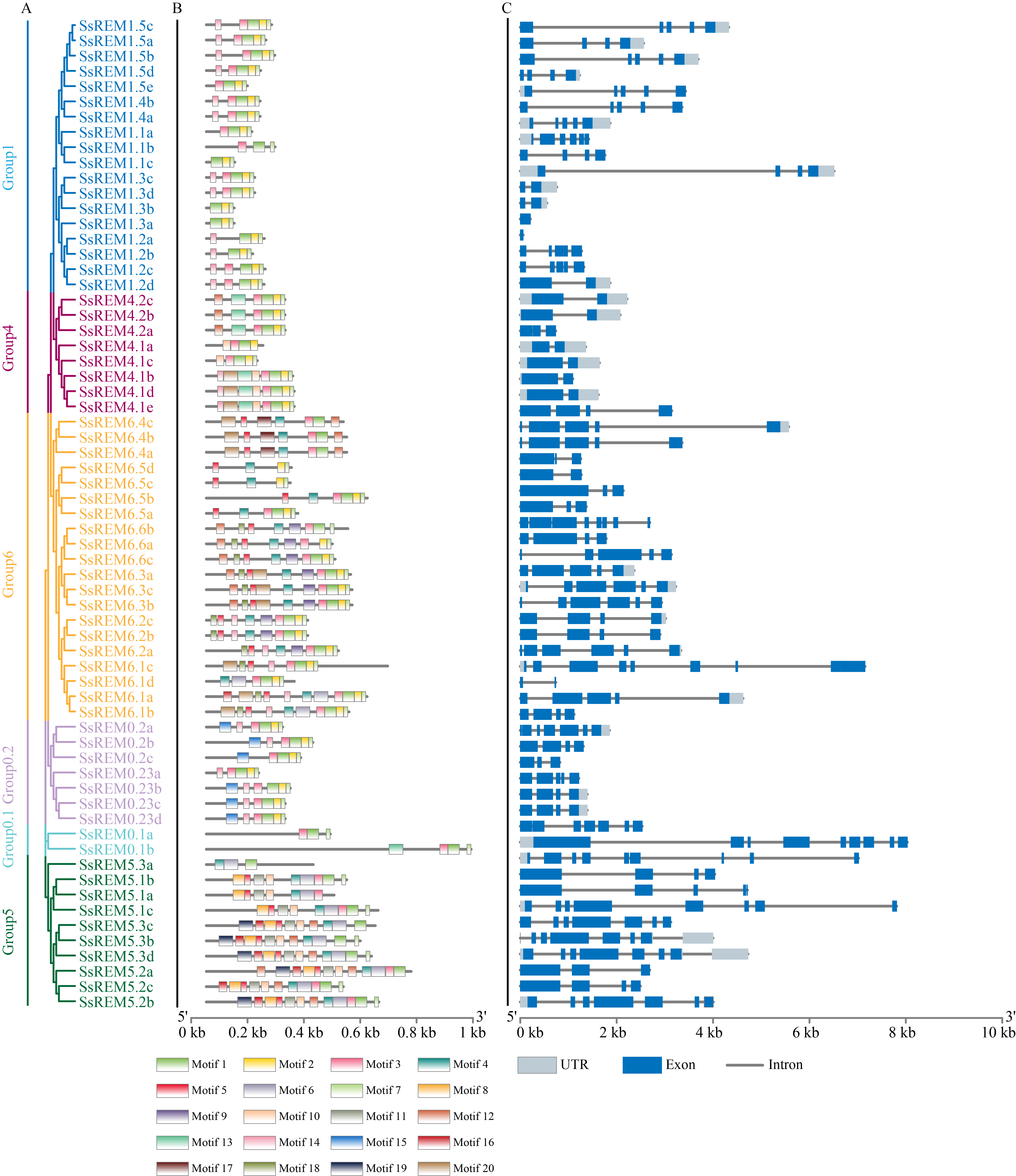

Supplement: Supplementary Figure 1 — Phylogenetic relationships, conserved motifs and structure analysis of the Remorin (SsREM) genes family. (A) The phylogenetic tree of Remorin proteins in Saccharum spontaneum. (B) Conserved motifs analysis of the Remorin genes family. (C) Gene structure analysis of the Remorin genes family. The groupings of clusters are shown in different colors. Conserved motifs of Remorin proteins are indicated by differently colored boxes. Offwhite boxes represent UTR (untranslated region), dark blue boxes represent exon, black line represent intron. [file Image_1.jpeg]

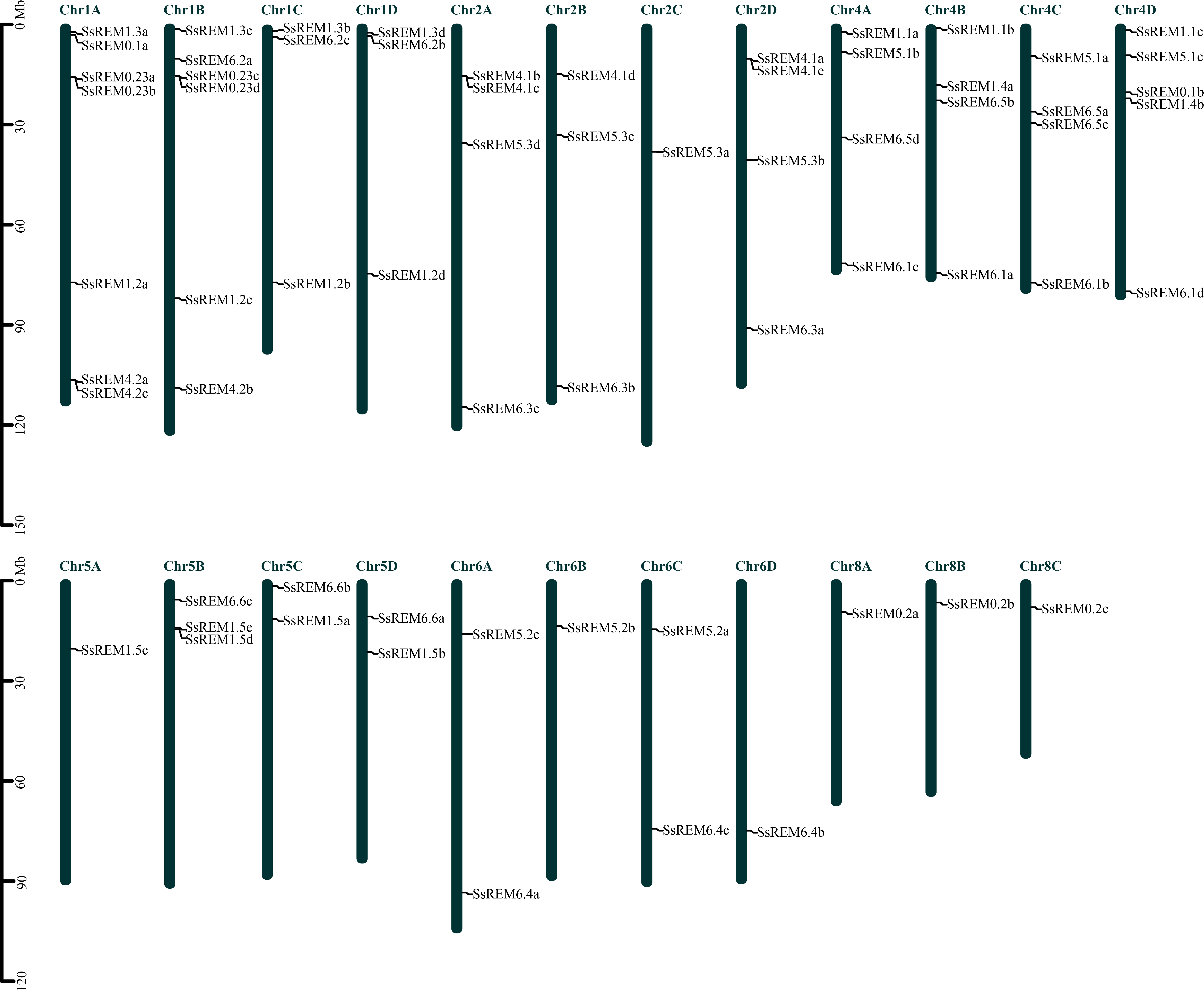

Supplement: Supplementary Figure 2 — Chromosomal locations of Remorin (SsREM) genes in the Saccharum spontaneum genome. The distributions of the 65 Remorin genes were determined according to the scaffold number and are shown in red. The numbers on the top indicate each chromosome number and the genome. [file Image_2.jpeg]

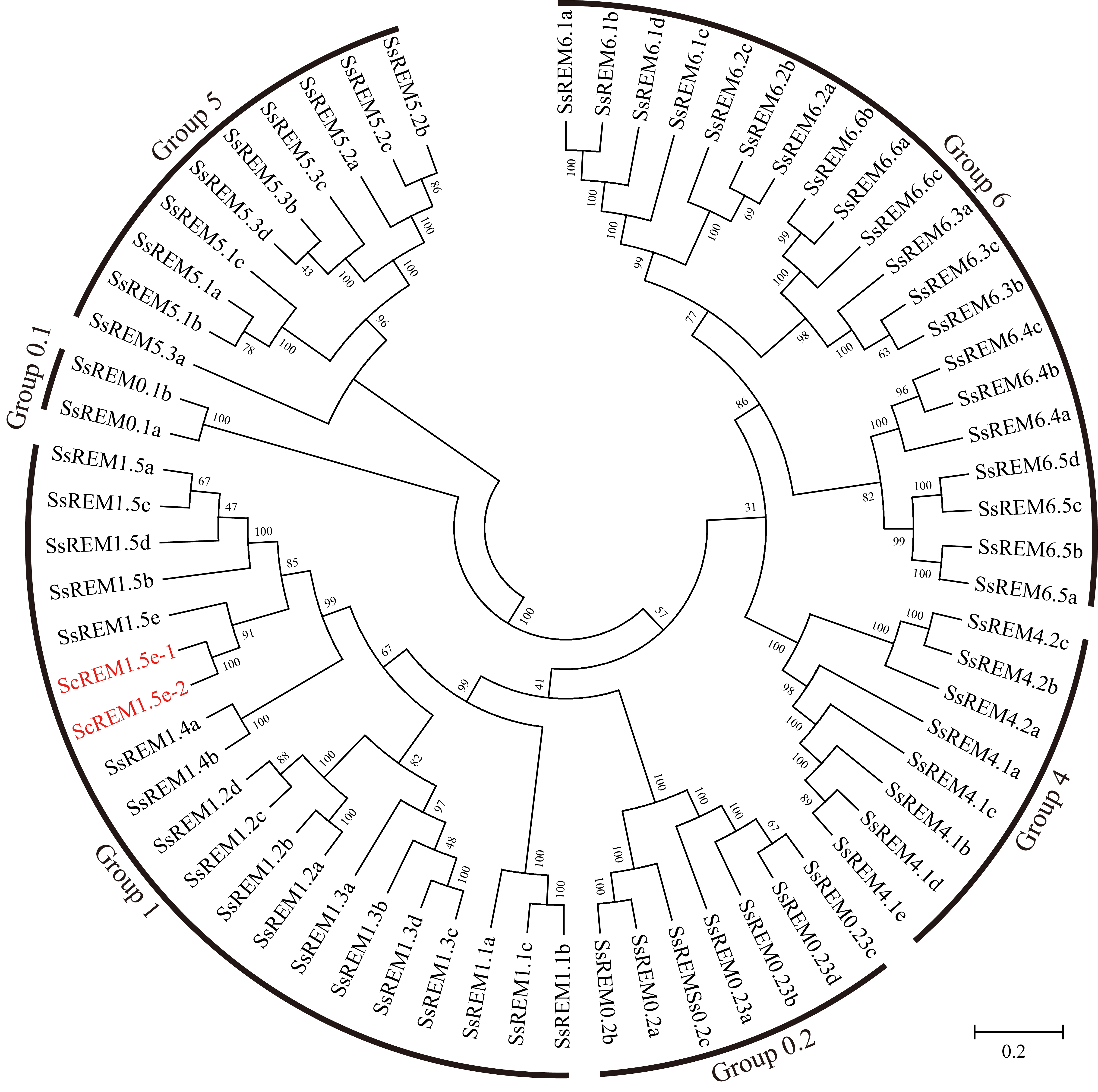

Supplement: Supplementary Figure 3 — Evolutionary tree of the two genes of sugarcane cultivar ROC22 with 65 SsREM genes. The two genes of sugarcane cultivar ROC22 were highlighted by the “leaves” in red, indicated that it belongs to REM1.5e branch. [file Image_3.jpeg]

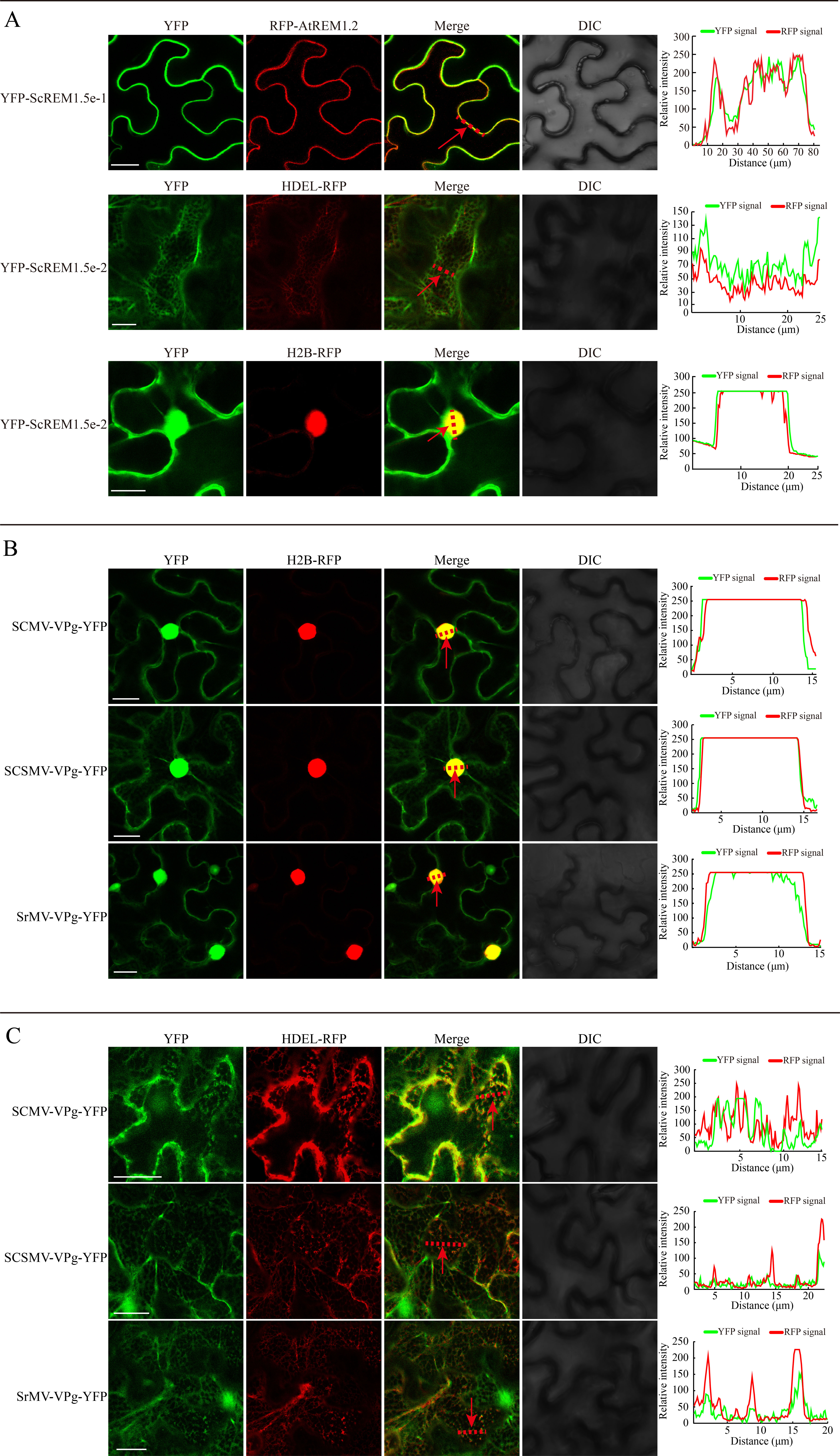

Supplement: Supplementary Figure 4 — Subcellular localization of ScREM1.5e-1/-2 or SCMV-/SCSMV-/SrMV-VPg in the epidermal cells of Nicotiana benthamiana. (A) Subcellular localization of YFP-tagged ScREM1.5e-1/-2 in N. benthamiana leaf epidermal cells. Bars = 20 μm. (B, C) Subcellular localization of YFP-tagged SCMV-/SCSMV-/SrMV-VPg in N. benthamiana leaf epidermal cells. The fifth column shows overlapping fluorescence spectra analysis of YFP and RFP signals marked in red dashed line. Bars = 20 μm. [file Image_4.jpeg]
